# The integration of single-cell RNA sequencing and spatial transcriptomics reveals the tumor microenvironment and spatial organization of testicular diffuse large B-cell lymphomas

**DOI:** 10.1016/j.gendis.2024.101475

**Published:** 2024-11-30

**Authors:** Xiaolong Wu, Jie Shi, Mujun Lu, Damin Yun, Sheng Gao, Longfei Hu, Fei Sun

**Affiliations:** a Department of Urology and Andrology, Sir Run Run Shaw Hospital, Zhejiang University School of Medicine, Hangzhou, Zhejiang 310016, China; b Institute of Reproductive Medicine, Nantong University School of Medicine, Nantong, Jiangsu 226001, China; c Department of Urology and Andrology, Ren Ji Hospital, School of Medicine, Shanghai Jiao Tong University, Shanghai 200001, China; d Singleron Biotechnologies Ltd., 211 Pubin Road, Nanjing, Jiangsu 210061, China

**Keywords:** CREB, E2F, Primary testicular diffuse large B-cell lymphoma (PT-DLBCL), Single-cell RNA-seq, Spatial transcriptomics

## Abstract

Primary testicular diffuse large B-cell lymphomas (PT-DLBCL) are a collection of 1%–9% of testicular tumors. However, the characterization of the tumor microenvironment and spatial organization of PT-DLBCL is poorly understood. We profiled the transcriptomes of 19,559 single cells derived from a PT-DLBCL patient via single-cell RNA sequencing. We found that the tumor microenvironment was majorly composed of three exhausted CD8^+^ T cell subpopulations and two B cell subpopulations, and the genetic heterogeneity was further analyzed. Then, transcription factors related to PT-DLBCL cell proliferation and development were identified. Our results demonstrated that inhibiting E2F and CREB could decrease cell proliferation, induce apoptosis in human B-lymphoma cells, and inhibit tumor growth in xenograft testicular DLBCL models. Subsequently, chromatin immunoprecipitation sequencing was performed to identify the enriched loci of E2F and CREB that regulate human B-lymphoma cell proliferation and apoptosis. To annotate the precise spatial cellular composition of testicular DLBCL, we performed spatial transcriptomics. The spatial organization of PT-DLBCL, especially the spatial location of exhausted CD8^+^ T and B cells, was identified. Concurrently, we delineated the expression patterns of key genes, including MALAT1, RPS3A, RPS7, RPS23, RPS27A, IGHM, HINT1, and HSPA8, across various regions. In this study, we unveiled the spatial architecture of the tumor microenvironment in DLBCL, where exhausted T cells were strategically positioned around tumor B cells, and macrophages, in turn, encircled the exhausted T cells. Inhibition of E2F and CREB in the tumor microenvironment may be a novel therapeutic option for testicular DLBCL patients.

## Introduction

Human spermatogenesis is a complex and highly coordinated process.^1^ It can be adversely affected by numerous factors such as obesity, diabetes, environmental chemicals, varicoceles, genetic factors, and tumors, all of which may contribute to infertility in males.^2^ As the most common solid malignancy, testicular tumors occur among adolescents and young males,^3^^,^^4^ and its incidence has reportedly increased in the past two decades.^5^^,^^6^ In 2016, the WHO stated that testicular tumors should be classified as testicular germ cell tumors, non-testicular germ cell tumors, and other rare non-germ cell tumors such as primary testis lymphomas.^7^ Primary testis lymphomas can be majorly categorized into three subtypes: Burkitt lymphomas, follicular lymphomas, and diffuse large B-cell lymphomas (DLBCLs). The latter condition is predominantly observed in patients older than 50 years. Primary testicular diffuse large B-cell lymphoma (PT-DLBCL) represents the most common subtype of testicular lymphoma. Patients with PT-DLBCL typically exhibit symptoms including a painless unilateral testicular mass, weight loss, night sweats, fever, and anorexia.^8^ DLBCLs, including activated B-cell-like, germinal center B-cell-like, and unclassified DLBCLs, have been shown to be phenotypically and genetically heterogeneous via gene expression profiling techniques.^9^^,^^10^

Advances in genomic technologies over the past few years have resulted in remarkable progress in understanding the pathogenesis of DLBCLs. DLBCLs are caused by oncogenic dysregulation, including somatically acquired gene copy number-related changes and non-silent point mutations, in a manner analogous to that observed for other human tumors.^11^ In addition, chromosomal translocations and aberrant somatic hypermutations are the two primary genetic damage mechanisms contributing to DLBCLs.^12^ For example, Schmitz et al discovered four prominent genetic subtypes of DLBCL via exome and transcriptome sequencing, targeted amplicon resequencing, and array-based DNA copy number analysis of 574 DLBCL biopsy samples.^13^ Moreover, the tumor microenvironment (TME) in B cell lymphoma is multifaceted, encompassing blood vessels, a diverse array of immune cells, extracellular matrix components, and stromal cells. Notably, the spatial arrangement of these distinct cell types may contribute to the metastasis and proliferation of malignant cells. PT-DLBCL occurs in an immune-privileged site within the testis, distinguishing it from other types of DLBCLs. The pathogenesis of PT-DLBCL remains not fully elucidated.^14^

Recently, advanced technologies have been used in scientific research, especially to study cell and tumor development. Single-cell RNA sequencing (scRNA-seq) is a powerful technique that can be used to sequence and analyze thousands of diverse cells, including immune, stromal, and malignant subsets, in a single-cell resolution.^15^ In 2019, Noemi Andor et al performed the scRNA-seq of follicular lymphomas. Their findings offer fresh perspectives on the microenvironment of follicular lymphomas, detailing both malignant B-cell varieties and immune subpopulations. Such insights could pave the way for innovative approaches to follicular lymphoma treatment.^16^ Notably, scRNA-seq has been used successfully to study the genomic and transcriptomic landscape of single cells. Spatial information regarding the tissue context and subcellular location can be derived based on high-throughput measurements because of the complexity of multicellular organisms.^17^ To address the problems associated with the disadvantages of scRNA-seq, Patrik et al developed spatial transcriptomic methods to provide quantitative gene expression data and visualize mRNA distribution within tissue sections.^18^ So far, spatial transcriptomics has been used in studies on prostate cancer,^19^^,^^20^ breast cancer,^21^ bone marrow niche organization,^22^ liver physiology, and disease biology.^23^

Here, we combined scRNA-seq and spatial transcriptomics to reveal the tissue architecture of PT-DLBCL. We clarified that the TME majorly consisted of three CD8 exhausted T cell subpopulations and two B cell subpopulations and further analyzed their genetic heterogeneity. The down-regulation of E2F and CREB could inhibit B-lymphoma cell proliferation, promote cell apoptosis, and inhibit tumor growth in xenograft testicular models of DLBCL. We preliminarily elucidated E2F and CREB regulation mechanisms in human B-lymphoma cells. Finally, we performed spatial transcriptomics to draw the spatial organization of PT-DLBCL and show the occurrence of the same cell-type aggregated growth.

## Materials and methods

### Preparation of single-cell suspensions

Tumor and paracancerous tissues were obtained from a 76-year-old PT-DLBCL patient. The patient presented with a firm, 3 cm seized, painless mass in the left without right testis involvement, with no treatment before surgery. The pathological type was diagnosed as PT-DLBCL. The sample was obtained in the Department of Urology and Andrology, Ren Ji Hospital, School of Medicine, Shanghai Jiao Tong University (Shanghai, China) with the patient's consent for research (IRB approved protocol: TDLS-2020-36). All two fresh tissue samples were collected in the GEXSCOPE Tissue Preservation Solution (Singleron Biotechnologies: https://singleron.bio/product/detail-8.html)^24^ at 2 °C–8 °C. Prior to tissue dissociation, the specimens were washed with Hanks Balanced Salt Solution three times and minced into 1–2 mm pieces. The tissue pieces were digested in 2 mL GEXSCOPE Tissue Dissociation Solution (Singleron Biotechnologies) at 37 °C for 15 min in a 15 mL centrifuge tube while continuously agitating the contents. Following digestion, a 40-micron sterile strainer (Corning) was used to separate cells from cell debris and other impurities. The cells were centrifuged at 1000 rpm at 4 °C for 5 min, and cell pellets were resuspended into 1 mL phosphate buffer saline (HyClone). To achieve red blood cell removal, 2 mL of GEXSCOPE Red Blood Cell Lysis Buffer (Singleron Biotechnologies) was added to the cell suspension, and the contents were incubated at 25 °C for 10 min. The mixture was centrifuged at 1000 rpm for 5 min, and the cell pellet was resuspended in phosphate buffer saline. Cells were counted using the TC20 automated cell counter (Bio-Rad). The concentration of the single-cell suspension was adjusted to 1 × 10^5^ cells/mL in phosphate buffer saline. Then, 8000–9000 cells per sample were targeted on the Chromium platform (10× Genomics). Single-cell mRNA libraries were built using the Chromium Next GEM Single Cell 3′ Library Construction V3 Kit. The resulting single-cell RNA-seq libraries were sequenced on an Illumina NovaSeq instrument to generate 150-bp long paired-end reads.

### Processing and quality control of scRNA-seq data

Raw sequencing data were converted to FASTQ files aligned to the human genome's reference sequence (assembly: GRCH38) using the Cell Ranger 3.0.2 pipeline (10× Genomics). The human genome and gene annotations were downloaded from Ensembl genome browser 98 and processed using the mkref command from the Cell Ranger 3.0.2 (10× Genomics) pipeline. The reference sequence was then used in the Cell Ranger “count” command to generate expression matrices. We designated testicular samples obtained from three healthy males that were used to generate scRNA-seq data as the normal sample (GEO: GSE120508).^25^ The gene expression matrix was then processed and analyzed using the Seuratv3 package.^26^ To filter out low-quality cells, we first removed cells for which less than 100 genes or less than 200 unique molecular identifiers (UMIs) were detected and removed over 20 % of the genes derived from the mitochondrial genome.

### Dimensionality reduction, clustering, cell-type labeling, and visualization

Seurat v3 was used for dimensionality reduction, clustering, and visualization.^26^ We used the filtered expression matrix for each sample dataset to identify cell subsets. The filtered gene expression matrix was normalized using Seurat's Normalize Data function, in which the number of UMIs of each gene was divided by the sum of the total UMIs per cell, multiplied by 10,000, and then transformed to the log scale (ln(UMI-per-10000 + 1)). After data were normalized, highly variable genes were identified and used for principal component analysis. Subsequently, clustering was performed with 20 principal components and a resolution of 1.0 via graph-based clustering and visualized using t-distributed stochastic neighbor embedding (t-SNE) or uniform manifold approximation and projection (UMAP) with Seurat functions such as RunTSNE and RunUMAP. For clustering T lymphocytes, myeloid cells, endothelial cells, and B cells, the top 20 principal components were selected with resolution parameters of 0.8, 0.6, 0.6, and 0.6, respectively. CellCycleScoring (Seurat software) was used for the cell cycle phase positioning of each cell, cells were colored by cell cycle score. For T cell subpopulation identification, the relationship between constructed gene expression values and S and G2M cell cycle scores was performed based on the ScaleData function (Seurat software). The residuals of the model represent a corrected expression matrix for downstream dimensionality reduction. Cell types were annotated using the SynEcoSys database (Singleron Biotechnologies).

The method helped to identify cell clusters that were then visualized on the UMAP space using the DimPlot command. Cell-type-specific canonical gene markers were used to label clusters that differentially expressed those markers. The Wilcox test was performed to identify differentially expressed genes for each cluster and accurately label individual clusters. We used the FindAllMarkers function in Seurat to obtain a list of differentially expressed genes for each cluster. Gene expression was visualized using the FeaturePlot, DoHeatMap, and VlnPlot functions of Seuratv3.

### Xenograft testicular DLBCL model construction and the treatment of animals

After obtaining approval from Run Run Shaw Hospital, Zhejiang University School of Medicine (approval number: 20210825–30), animal experiments were conducted according to the institutional guidelines for the use of laboratory animals. Eight-week-old male severe-combined-immune-deficiency mice were raised in a specific pathogen-free barrier system and fed sterilized food and water *ad libitum*. The mice were weighed and intraperitoneally injected with 100 mg/kg of ketamine and 10 mg/kg of xylazine mixture solution. Anesthetized mice were transferred to a thermally controlled platform and maintained at 37 °C prior to surgery. After shaving the fur on the abdomen with scissors, the skin was disinfected three times with iodophor cotton balls. The incision was located approximately 1.5 cm above the penis and was perpendicular to the midline of the abdomen. A surgical blade was used to cut a 1 cm incision. After opening the abdominal cavity of the mice, sterile smooth forceps were used down the left buttock, and the fatty tissue and the testis were pulled out carefully. The testis was maintained stably, and using a pipette, 10 μL of cell suspension was accurately quantified and transferred to an injection needle. After administering the injection, the muscles and skin were closed with absorbable sutures. The mouse activity and testicular cancer growth were observed daily after inoculation. Mice were randomly divided into two groups on the seventh day after surgery. One group was injected with an inhibitor of E2F (20 μM) and CREB (10 μM) *in situ* in the testis, and the other group was injected with DMSO twice weekly. The mice were euthanized via CO_2_ inhalation after 20 days, in accordance with the AVMA Guidelines for the Euthanasia of Animals. All involving animal experiments were approved by the Ethics Committees of Nantong University (No. S20210401-901).

### Spatial RNA-seq data processing, integration, and visualization

Spatial RNA-seq data obtained from two 10× Visium capture areas, in which each area contained 5000 barcoded spots, were first processed by Space Ranger analysis software. The resultant matrix files were analyzed using the Seurat v4.0.1 package.^27^ To optimize spatial clustering, BayesSpace (v1.2.0) was applied for spatial transcriptomic data.^28^ Spatial transcriptomic raw counts were preprocessed by spatial preprocess function with log-normalization and spatial variable gene selection. Spatial clustering was performed based on the top 15 principal components with the initial clustering method setting as kmeans. To estimate spatial cell type composition, spatial transcriptomics deconvolution was started with Seurat label transforming. Data with cell types annotated from scRNA-seq and spatial RNA-seq data were subjected to the re-normalization and principal component analysis by Seurat using SCTransform and RunPCA. Then, FindTransferAnchors was performed to find anchors for integration with the two datasets. The underlying composition of cell types was predicted to deconvolute each of the spatial voxels by the TransferData method. Through the steps above, a table containing the proportion of cell types in each spot from spatial data is available. This table was passed to stLearn,^29^ and used to do the deconvolution visualization and to quantify the cell type composition in each cluster. Hotspot (v0.9.0)^30^ was used to identify spatial gene modules that illustrate heterogeneity within tissue sections. Briefly, we used the “danb” model and built latent space according to spatial coordinates with the number of neighbors set to 300. The top 500 genes with the highest autocorrelation z-score were selected for module identification. Modules were then identified using the create_modules function, with min_gene_threshold = 15 and fdr_threshold = 0.05. Module scores were calculated using the calculate_module_scores function. Spatial transcriptomics assays can help to profile spatial regions in tissue sections but do not have single-cell resolution. CellTrek (https://github.com/navinlabcode/CellTrek) was used to achieve single-cell spatial mapping through the co-embedding and metric learning approaches.^27^ Gene expression can be projected to spatial tissue sections and visualized using SpatialDimPlot and SpatialFeaturePlot in Seurat.

### Statistical analysis and plots

Wilcoxon tests were conducted using the R language to compare the differences between two samples. *p* values < 0.05 were considered statistically significant. R language was applied to plot heatmaps (using the pheatmap package), box plots, bar plots, *etc* (using the ggplot2 package).

## Results

### Identifying cell population in paracancerous and cancerous tissues via scRNA-seq analysis

**Figure 1:**
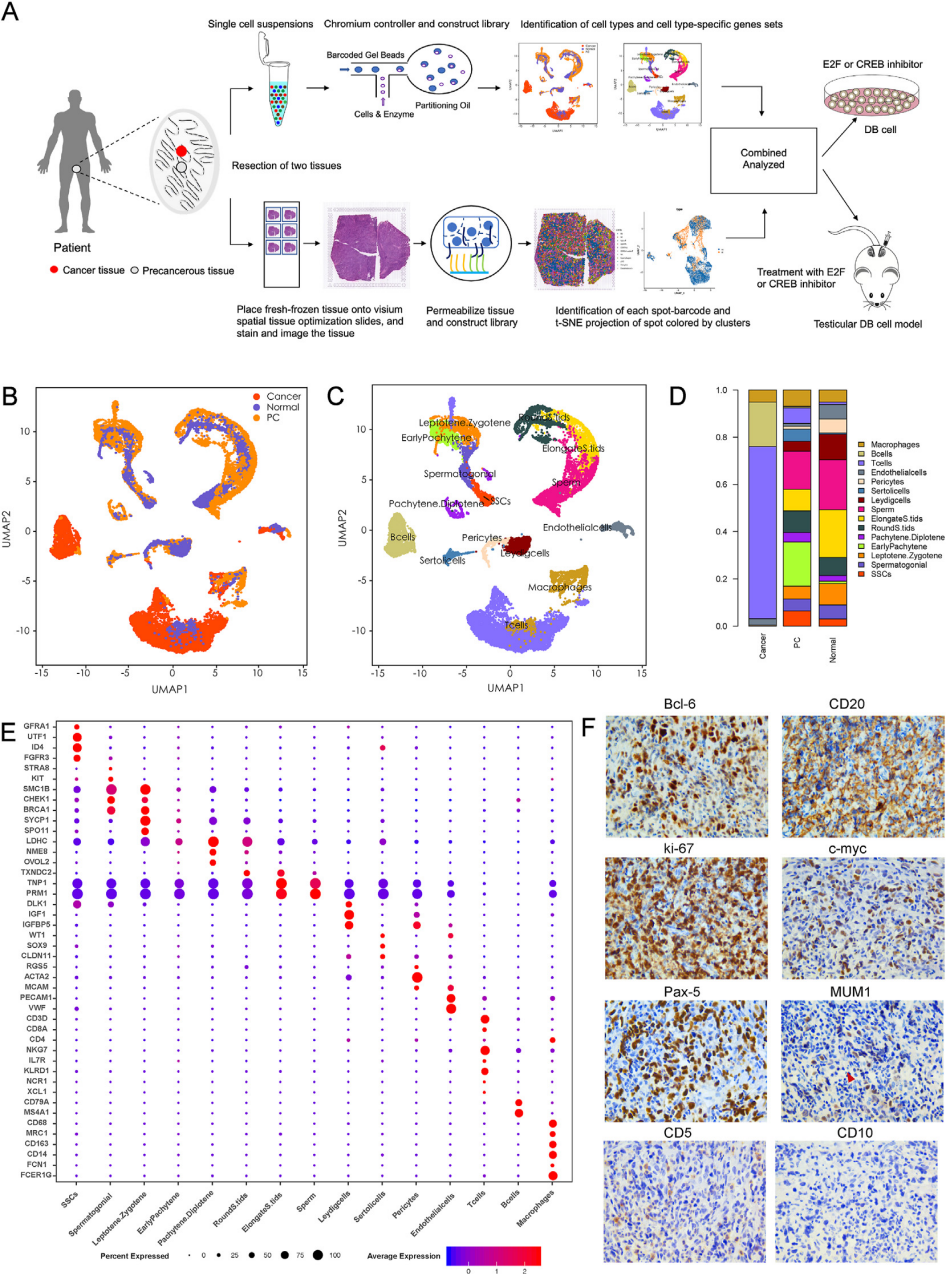
Single-cell RNA-sequencing analysis of normal human testis, paracancerous, and cancer tissues obtained from a patient with testicular DLBCL. **(A)** Schematic of the experimental design and analysis. Paracancerous and cancer tissues obtained from surgically resected DLBCLs were split and processed for scRNA-seq and spatial transcriptomics. **(B)** Single-cell expression of all cells from healthy, paracancerous, and testicular DLBCL tissues. The red, blue, and orange colors represent cancerous, healthy, and paracancerous cells, respectively. **(C)** UMAP plot of cells from Figure 1B. Cell types: germ cells, B cells, Sertoli cells, pericytes, Leydig cells, macrophages, T cells, and endothelial cells. **(D)** Percentages of different cell types in cancerous, paracancerous, and normal cells. **(E)** Representative gene expression for each cell type. **(F)** Immunohistochemical staining of primary testicular DLBCL. (+++) represents high expression; (++) represents mild expression; (+) represents low expression; and (−) represents negative expression.

We first processed freshly obtained paracancerous and cancerous tissues from a PT-DLBCL patient who had not received treatment and was undergoing surgery. These tissues were parallelly used for scRNA-seq and spatial transcriptomic analysis (Fig. 1A). Meanwhile, we combined the public domain scRNA-seq datasets of three healthy male testes, considered as a normal control group, with our data for further analysis.^25^ We merged the datasets of the three groups for analysis (Fig. 1B). Following rigorous quality control measures, 23,540 single cells were identified and included in the downstream analysis of this study. We identified cell clusters corresponding to the 15 major cell types (Fig. 1C) defined by the known cell-type markers shown in Figure 1E. Next, Figure 1D shows the percentages of the 15 major cell types in the three groups. Notably, cancer tissues mainly consisted of pericytes (∼1%), endothelial cells (∼3%), T cells (∼70%), B cells (∼20%), and macrophages (∼6%). In comparison to cancer tissues, paracancerous tissues were composed of normal cells, including germ cells (∼76%), T cells (∼6%), B cells (∼1%), and other somatic cells (∼17%). Moreover, in healthy tissues, germ cells (∼72%) and somatic cells (∼25%) accounted for almost all the cell types, and there were few T cells (∼2%) and B cells (<1%). The cancer tissue was defined as PT-DLBCL tissue via scRNA-seq analysis. To confirm this, we performed the immunohistochemical staining of tumor samples that exhibited high expression levels of B-cell markers such as CD20. For the differential diagnosis of other lymphomas, the immunohistochemical staining of Bcl-6 (+++), ki-67 (+++), c-myc (+), Pax-5 (++), MUM1 (+), CD5(+), and CD10 (−) was performed (Fig. 1F). The results showed that the PT-DLBCL microenvironment was mainly composed of T cells, B cells, macrophages, and endothelial cells, which were substantially different from healthy and paracancerous tissues.

### Testicular DLBCL-specific immune microenvironment

**Figure 2:**
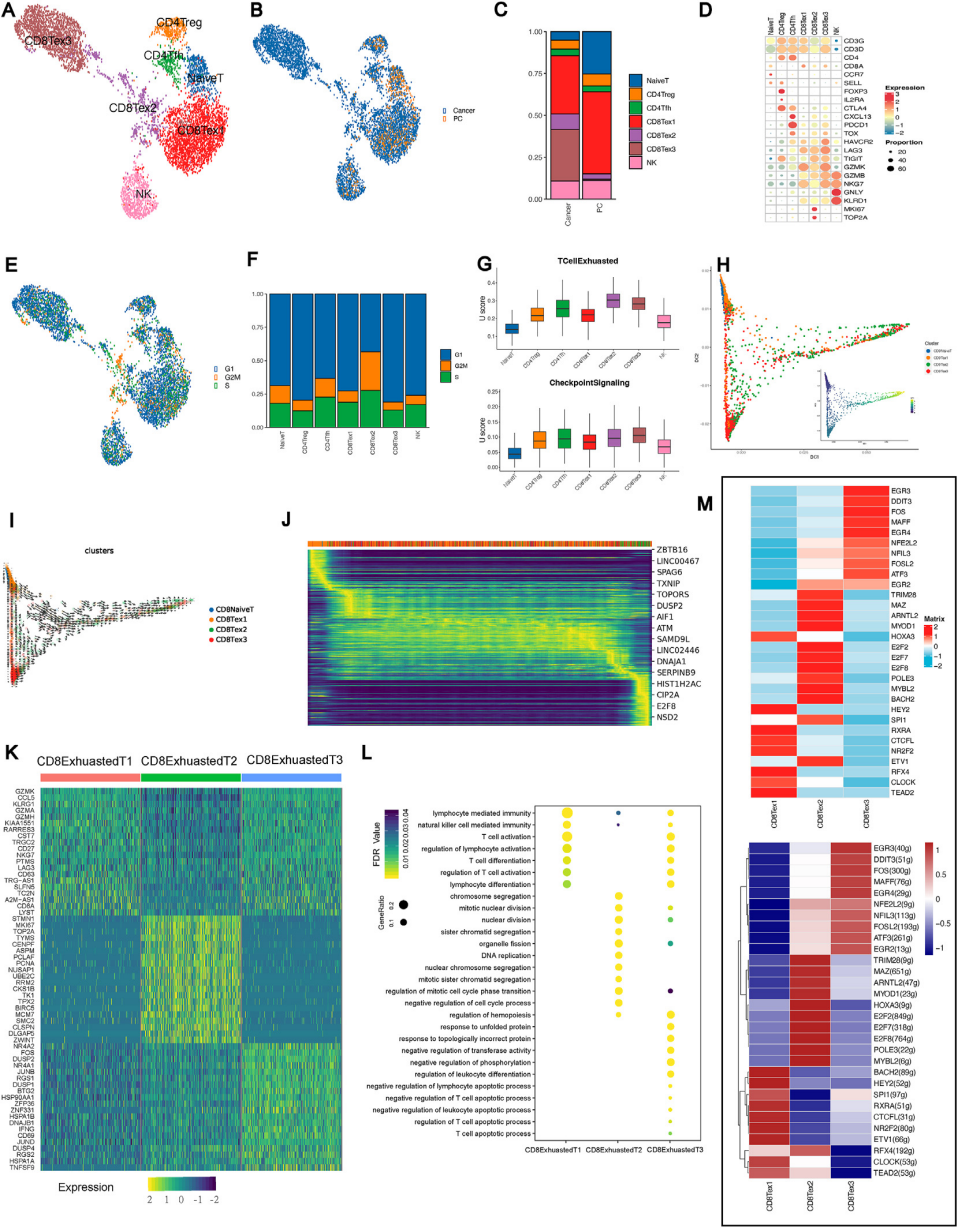
Immune cell atlas of the testicular DLBCL microenvironment at single-cell resolution. **(A)** Clustering visualization of T cell subsets based on single-cell RNA-sequencing. Cell types are color-coded: CD8Tex1 (red), CD8Tex2 (purple), CD8Tex3 (brown), CD4 Treg (orange), CD4 Tfh (green), naïve T (blue), and NK cells (pink). **(B)** The UMAP plot displaying the distribution of cell states in cancer (blue) and pre-cancerous (orange) conditions. Each point represents an individual cell. **(C)** The bar graph showing the proportion of each T cell subset within cancer and pre-cancerous conditions, indicating the distribution of naïve T, CD4 Treg, CD4 Tfh, CD8Tex1, CD8Tex2, CD8Tex3, and NK cells. **(D)** The dot plot displaying the expression levels of marker genes across T cell subsets. The color intensity reflects gene expression (red for higher and blue for lower expression), and the dot size indicates the proportion of cells expressing the gene. **(E)** The UAP plot illustrating the distribution of cell cycle phases among single cells. Cells are categorized into different cell cycle phases: G1 phase (blue), G2/M phase (orange), and S phase (green). **(F)** The bar chart representing the proportion of immune cell types in each phase of the cell cycle: G1 (blue), G2/M (orange), and S (green) phases. **(G)** The box plots representing the exhaustion levels (top) and checkpoint signaling (bottom) across different T cell subsets, quantified by U scores. **(H)** The developmental trajectory of CD8 T cells is inferred by the diffusion map. **(I)** RNA velocity of each CD8 T cell subtype. **(J)** The heatmap illustrating the dynamics gene expression profile during CD8 naïve T-to-exhausted CD8 T differentiation. **(K)** Heatmap of three subsets of exhausted CD8^+^ T cells. Top, type of exhausted CD8^+^ T cells; left, gene symbol. **(L)** Dot plot of enriched Gene Ontology terms for highly expressed genes in the three subsets of exhausted CD8^+^ T cells. Left, GO term; below, type of exhausted CD8^+^ T cells. **(M)** Heatmap of transcription factor regulatory activity (lower) and transcription factor expression (upper).

To characterize the immune microenvironment of immune cells in the TME of testicular DLBCLs, we classified T and natural killer (NK) cells into 7 clusters to explore the inherent heterogeneity of the immune microenvironment, and the UMAP plot shows the distribution among samples. This analysis revealed seven distinct immune cell clusters: naïve T cells, CD4^+^ regulatory T cells (Tregs), follicular helper T cells (Tfh), and three subsets of exhausted CD8^+^ T cells (CD8Tex1, CD8Tex2, CD8Tex3), along with NK cells (Fig. 2A). Figure 2B showed that the cell types of paracancerous tissue were mainly enriched in CD4Treg, CD4Tfh, naïve T, NK, CD8Tex1, and a few CD8Tex2 cells. However, cancer tissues consisted of numerous CD8Tex1, CD8Tex2, and CD8Tex3 cells. The percentages of cell types also illustrated the results shown above (Fig. 2C). Gene markers were used to identify different cell clusters.

Indeed, markers typically associated with T cell exhaustion, such as PDCD1, LAG3, and TOX,^31^ were found to be highly expressed in the subsets of exhausted CD8^+^ T cells identified as CD8Tex1, CD8Tex2, and CD8Tex3 (Fig. 2D). Figure 2E showed a nuanced dissection of cell cycle stages across diverse immune cell subsets. The UMAP visualization in Figure 2E delineates the cellular distribution within distinct phases of the cell cycle (G1, S, G2M), thereby highlighting the proliferative dynamism among the sampled immune populations (Fig. 2E). Notably, a substantial majority, approximately 57% of the CD8Tex2 cells, are captured in the mitotically active G2M and S phases (Fig. 2F). To probe the functionality of T cells in the TME, we assessed gene expression related to T cell exhaustion and checkpoint signaling in different immune cell types. A scoring system quantified exhaustion, which revealed minimal levels in naïve T cells and progressively higher levels in exhausted CD8^+^ T cell subsets. Checkpoint signaling varied across cells, with Tregs and Tfh cells showing moderate levels and exhausted CD8^+^ subsets mirroring NK cells' signaling profile (Fig. 2G). The differentiation trajectory indicates a progressive transition from a naïve state to an exhausted state among CD8^+^ T cells. CD8Tex1 is indicative of an intermediate exhaustion phase, whereas CD8Tex2 and CD8Tex3 demonstrate features more consistent with terminal exhaustion (Fig. 2H). This progression is characterized by an up-regulated expression of exhaustion markers and checkpoint signaling, as quantified in earlier analyses. RNA velocity analysis reveals the differentiation of CD8^+^ T cells into exhausted CD8^+^ T cells in the TME, with clear transitions between CD8^+^ naïve T and the various exhausted subsets (CD8Tex1, CD8Tex2, CD8Tex3) (Fig. 2H, I). Figure 2J illustrates a gene expression heatmap of the top genes from CD8^+^ T cells aligned with the differentiation trajectory.

CD8^+^ T cells play a crucial role against intracellular pathogens and tumors, thereby protecting the immune system. When exposed to persistent antigen and inflammatory signals, CD8^+^ T cells gradually result in the deterioration of T cells to an “exhaustion” state.^32^ We selected the top 20 highly expressed genes in CD8Tex1/2/3 cells, and the result showed that their expression patterns were entirely different (Fig. 2K) due to changes in the functions of CD8Tex1/2/3 cells. Gene ontology analyses were used to further investigate the functions of CD8Tex1/2/3 cells and identify whether the three subsets of exhausted CD8^+^ T cells were functionally enriched (Fig. 2L). The signaling pathway of CD8Tex1 cells was mainly enriched in “T cell activation”, “lymphocyte-mediated immunity”, and “T cell differentiation”, like that observed for healthy T cells. In comparison to CD8Tex1 cells, CD8Tex3 cells were enriched in the “negative regulation of pathways”, including “negative regulation of phosphorylation”, “negative regulation of the lymphocyte apoptotic process”, “negative regulation of the T cell apoptotic process”, and “regulation of the T cell apoptotic process”. However, the enriched signaling pathway in CD8Tex2 cells was distinctly different from that of CD8Tex1 and CD8Tex3 cells that exhibited a loss in normal T cell functions. The heatmaps portray the regulatory landscape of transcription factors within distinct CD8^+^ T cell subsets. The upper heatmap quantifies the transcriptional expression levels. The lower heatmap demonstrates transcription factor regulatory activity that is calculated based on the set of transcription factor-regulated genes, delineating unique patterns that elucidate the regulatory influence exerted by these transcription factors in the subsets CD8Tex1, CD8Tex2, and CD8Tex3 (Fig. 2M).

Our result revealed that transcription factors such as E2F2, E2F7, and E2F8 were highly expressed in the CD8Tex2 subset. The E2F transcription factor family plays a vital role in controlling the cell cycle and encoding tumor suppressor proteins. As an essential part of the cyclin-dependent kinase (CDK)–RB–E2F axis, E2F will become active and expressed after alterations occur in one or more critical components of this axis, inducing uncontrolled cell proliferation in virtually all cancers.^33^^,^^34^ Our trajectory analysis of CD4^+^ T cell subsets revealed distinct separations among Tfh, naïve T, and Treg cells, indicating unique developmental pathways within the CD4^+^ T cell lineage (Fig. S2A, B). Figure S2C presented a detailed heatmap of gene expression across these subsets, highlighting the varied transcriptional landscapes that characterize each group, with genes such as NR6A1, PPP3CA, and IL4I1 displaying subset-specific expression patterns. In addition, we analyzed the GO enrichment of CD4^+^ Tregs, CD4^+^ Tfh, and naïve T cells, and the results showed that these three cell populations seemed to have normal immune functions (Fig. S2D). Collectively, these data showed that the testicular DLBCL-specific immune microenvironment mainly consisted of exhausted CD8^+^ T cells, which could be classified into clusters of three different types and demonstrated their potential tumor progression-related functions.

### B cell heterogeneity in PT-DLBCL and paracancerous tissues

**Figure 3:**
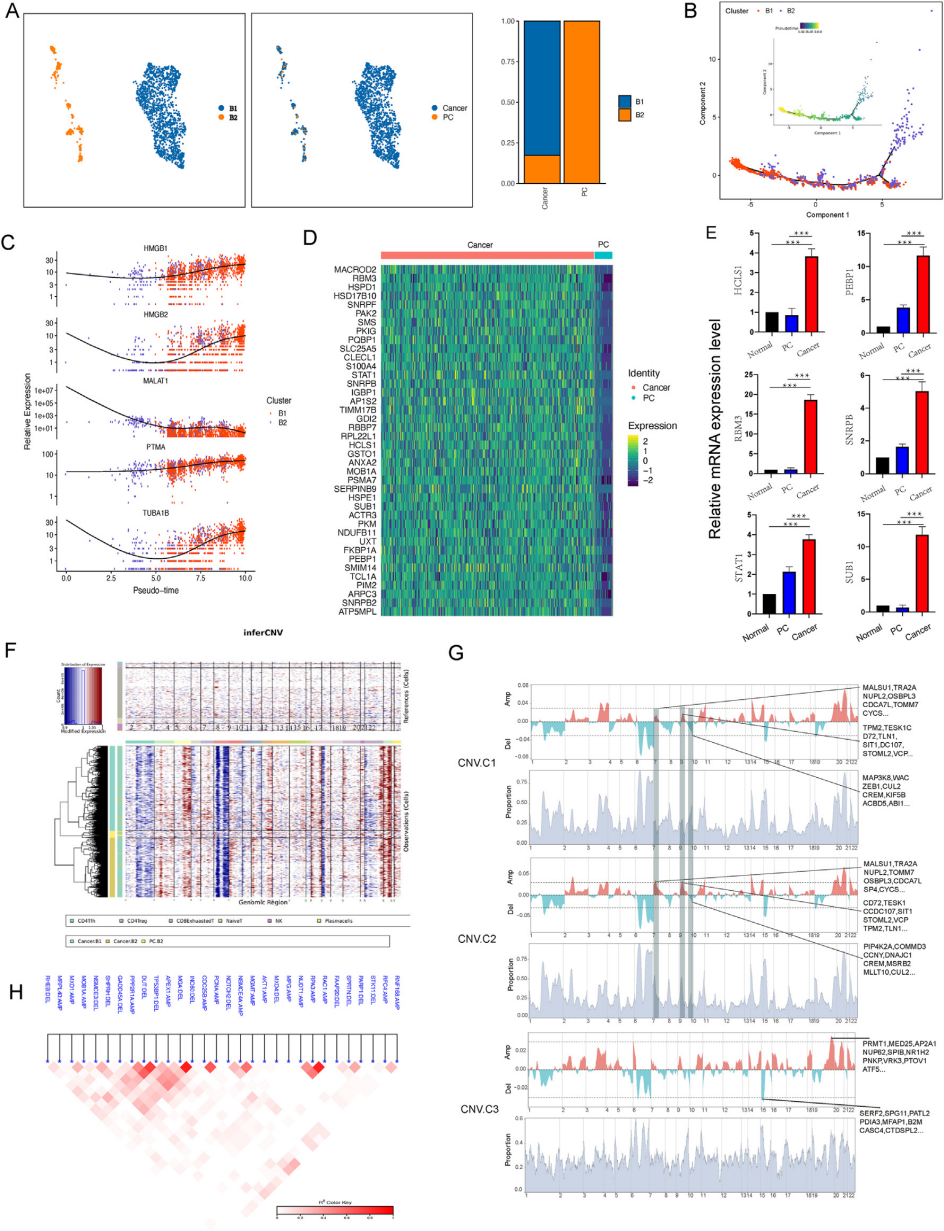
B cell atlas of the testicular DLBCL microenvironment at single-cell resolution. **(A)** The UMAP plot of the B cells showing 2 cell clusters; and the sample origin of the cells (indicated by labels and colors). **(B)** Pseudotime trajectory analysis of B cells. **(C)** The expression pattern of key genes along the pseudotime axis. **(D)** Differentially expressed genes of the top 40 genes (rows) that are differentially expressed in cancer and paracancerous tissues. **(E)** Gene expression levels of B cells in cancer, paracancerous tissues, and healthy testis tissues. **(F)** inferCNV shows the copy number variations of various cell types. **(G)** CNV shows the amplification and deletion of genes in CNV.C1, CNV.C2, and CNV.C3 cells. **(H)** Phylogenetic analysis shows an association between the amplification or deletion events for the same cells.

To analyze B cell heterogeneity in PT-DLBCL and paracancerous tissues, we independently re-clustered B cells from the scRNA data. The results showed that B cells were entirely different in these two tissues. Small amounts of normal B cells (B2) occurred in paracancerous tissues; however, PT-DLBCL contained a large number of heterogeneous B cells (B1) and a few B2 cells (Fig. 3A). Next, pseudotime trajectory analysis revealed a linear trajectory of B1 and B2 cells; B2 cells gradually differentiated into B1 cells (Fig. 3B). We further analyzed gene expression patterns to characterize the heterogeneity between B1 and B2. The results showed that HMGB1, HMGB2, PTMA, and TUBA1B were gradually up-regulated when B2 cells were differentiated into B1 cells (Fig. 3C). It was likely that HMGB1, which plays an active role in the TME, promoted the proliferation of DLBCL cells via the activation of AKT.^35^ Massimo et al reported that PTMA was localized in healthy human testes but was highly expressed in classic seminoma and Leydig cell tumors.^36^ In hepatocellular carcinomas, increased TUBA1B expression levels indicated a poor prognosis and resistance to chemotherapy.^37^ The heatmap showed that 33 genes that might contribute to PT-DLBCL progression were highly expressed in cancer tissue B cells (Fig. 3D). Furthermore, the presence of six genes (*HCLS1*, *PEBP1*, *RBM3*, *SNRPB*, *STAT1*, and *SUB1*) was verified by quantitative PCR in five different PT-DLBCL tissues, and the results were consistent with the bioinformatics data (Fig. 3E). Next, we identified large-scale copy number variations (CNVs) in B cells in cancerous tissues, and paracancerous tissues using an approach described previously.^38^ Based on CNV analysis, B cells were divided into 3 clusters (CNV1, CNV2, and CNV3). CNV3 cluster was paracancerous B cells, and CNV1 and CNV2 clusters were cancerous tissue B cells. The CNV pattern in cancerous B cells was largely different from that of paracancerous B cells (Fig. 3F). For example, some genes on chromosomes 7 and 9, including *MALSU1*, *TRA2A*, *NUPL2*, *OSBPL3*, *CDCA7L*, and *TOMM7 CYCS*, exhibited reduced levels of amplification. In contrast, there were increased deletions of some genes, including *TPM2*, *TESK1*, *CD72*, *TLN1*, *SIT1*, *DC107*, *STOML2*, and *VCP* (Fig. 3G). *OSBPL3* was reported to be involved in the development of certain human cancers, including gastric cancer^39^ and colorectal cancer.^40^ *STOML2* was significantly overexpressed in colorectal cancer and was associated with unfavorable prognoses.^41^ Subsequently, phylogenetic analyses showed an association between amplification or deletion events associated with the same gene (Fig. 3H). Interestingly, an INO80 deletion was strongly associated with the deletion of MGA. Belk et al demonstrated that INO80 and BAF chromatin remodeling could improve T cell persistence in tumors.^42^ MGA was predicted to enable DNA-binding transcription activator activity and was involved in cell fate specification and positive regulation of transcription by RNA polymerase II. MGA was found with loss-of-function mutations in lung cancers and colorectal cancers.^43^

These results showed that the B cell transcriptome and CNVs were vastly different between paracancerous and cancer tissues, and these modifications in the genes might have facilitated the progression of PT-DLBCLs.

### Down-regulation of E2F inhibits human B-lymphoma cell proliferation and facilitates cell apoptosis

**Figure 4:**
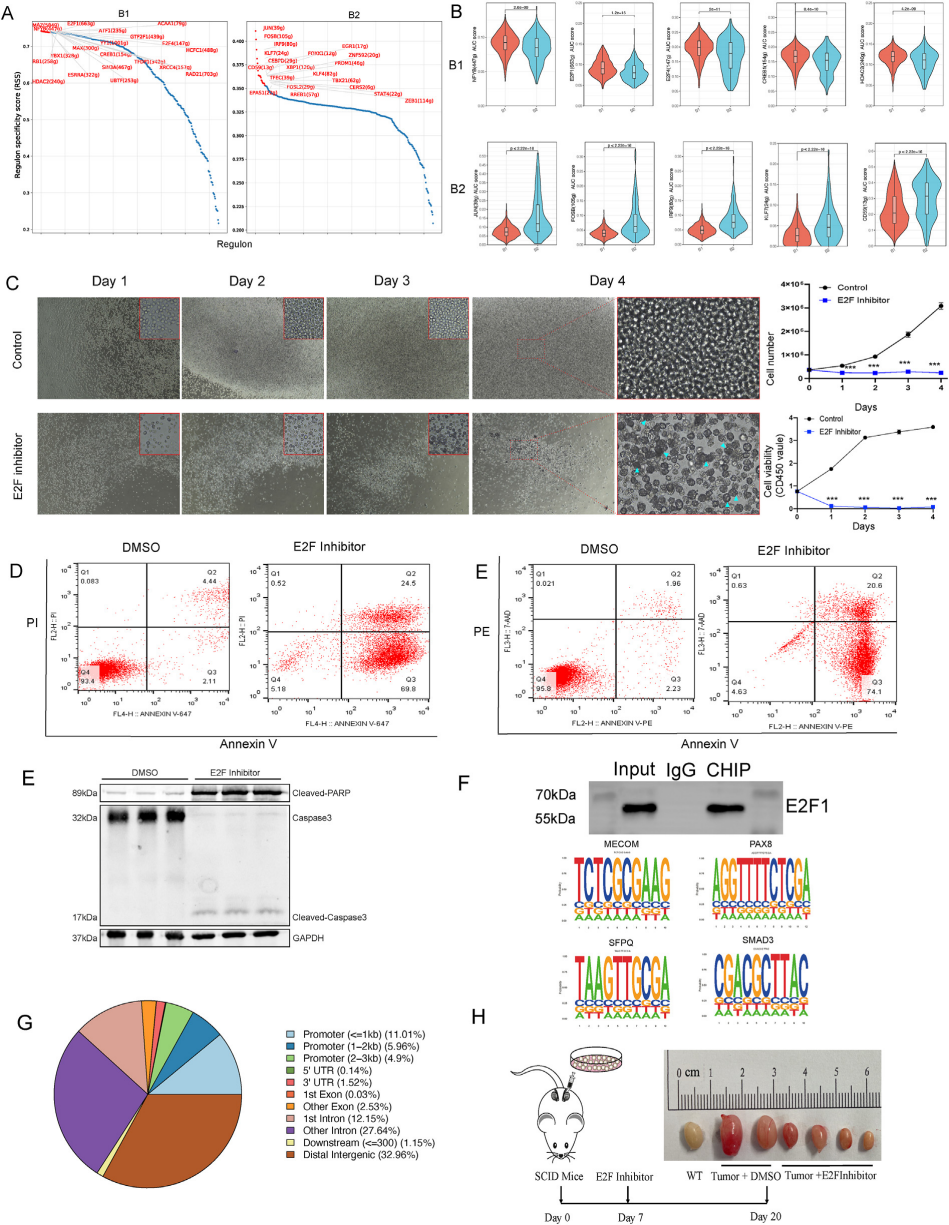
E2F inhibitor inhibited human B-lymphoma cell proliferation. **(A)** Regulon specificity score in B1 and B2 cells with top 20 specific labeled transcription factors. **(B)** The violin plots showing the distribution of high AUC scores of transcription factors in B1 and B2 cells. **(C)** Cell morphology under the microscope (left), cell number, and CCK8 levels indicate cell viability (right). **(D)** Flow cytometer analysis shows that human B-lymphoma cells underwent apoptosis after E2F inhibitor treatment. **(E)** Apoptosis protein levels of human B-lymphoma cells. **(F)** Western blot experiment demonstrates the efficacy of E2F antibody; the motifs of the top 4 E2F-binding genomic loci. **(G)** Percentages of E2F-binding genomic loci. **(H)** Treatment with E2F inhibitor for xenograft testicular DLBCL models.

To further identify critical regulators of B cells, we used the regulon specificity score to evaluate the activities of regulators associated with the B cells. We selected the most specific regulon scores associated with B1 and B2 cells (Fig. 4A), which were completely different. For example, *NYFB*, *E2F1*, *E2F4*, *CREB1*, and *HDAC2* were highly expressed in B1 cells compared with B2 cells; however, *Jun*, *FOSB*, *IRF9*, *KLF7*, and *CD59* were expressed at high levels in B2 cells (Fig. 4B). To explore the role of E2F, HLM006474, an E2F inhibitor, was added to human B-lymphoma cells *in vitro*. The results showed that proliferation was inhibited in human B-lymphoma cells on day 2 and the cell morphology was substantially altered on day 4 in the E2F inhibitor group (Fig. 4C). To identify whether cells with an altered morphology had undergone apoptosis, we performed annexin-PI/PE staining and flow cytometry (Fig. 4D, E). The results showed that 69.8% of the cells in the E2F inhibitor group were in the Q3 quadrant (annexin-V-positive, PI-negative), defined as early apoptotic cells and 24.5% were in the Q2 quadrant (annexin-V-positive, PI-positive), defined as late apoptotic cells, while 5.18% were in the Q4 quadrant (annexin V-negative, PI-negative), defined as normal cells. In contrast, 93.4% of the control group (DMSO) cells were in the Q2 quadrant and were healthy cells. To ensure the accuracy of the experiment, we also performed a flow cytometric analysis of annexin-PE-stained cells, and the results were consistent with those shown above. In summary, the results of this partial flow analysis showed that most of the cells in the E2F group were already in the early stage of apoptosis at day 3, which helped us to verify the result of apoptosis via Western blot. Tumor progression was strongly associated with E2F dysfunction, and E2F transcriptional activity was tightly regulated via the cell cycle throughout processes such as translational regulation, post-translational modification, protein degradation, binding to cofactors, and subcellular localization.^34^ We performed chromatin immunoprecipitation sequencing to identify the enriched loci of E2F. We initially verified the efficacy of the E2F1 antibody, as demonstrated in Figure 4F above. The results showed that E2F bound loci were enriched in *MECOM*, *PAX8*, *SFPQ*, and *SMAD3* (Fig. 4F bottom), which were mainly located in the promoter (21.87%), intron (39.79%), and intergenic (32.96%) regions (Fig. 4G). Wiggle plots for the four genes are presented in Figure S3. It was probable that MECOM was a transcriptional regulator and oncoprotein involved in cell differentiation and proliferation. In addition, Bleu et al reported that *PAX8* and *MECOM* could regulate ovarian cancer in tandem.^44^ SFPQ, an ABL1-binding protein, drives the development of B-cell acute lymphoblastic leukemia.^45^ We constructed a primary testicular human B-lymphoma cell tumor model of server-combined-immune-deficiency mice. A total of 15 mice were divided into three groups: 5 in the control group, 5 in the E2F group, and 5 in the CREB group. In the control group, 3 mice were successfully modeled, 4 in the E2F group, and only 2 in the CREB group (see methods). Our result showed that three groups of testes: the normal wild-type group has a typical size around 1 cm; the tumor control group shows enlarged testes due to tumor growth, with sizes up to 2 cm; and the E2F inhibitor treatment group demonstrates smaller tumors, with sizes appearing to be reduced towards the 1 cm range of the normal testis (Fig. 4H).

### Down-regulation of CREB inhibits human B-lymphoma cell proliferation and facilitates cell apoptosis

**Figure 5:**
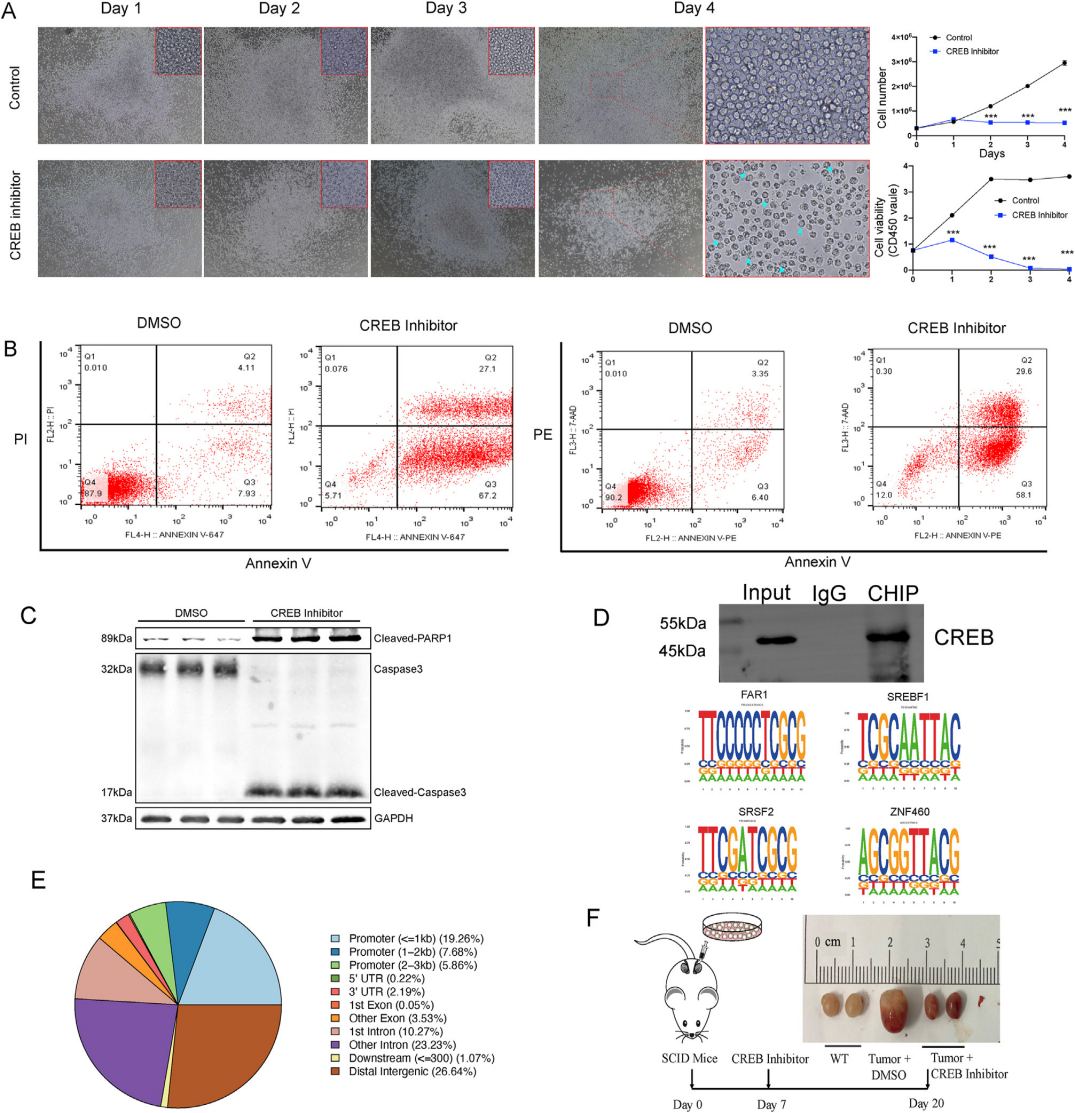
CREB inhibitor inhibited human B-lymphoma cell proliferation. **(A)** Cell morphology under the microscope (left), cell number, and CCK8 levels indicate cell viability (right). **(B)** Flow cytometer analysis showed that human B-lymphoma cells underwent apoptosis after CREB inhibitor treatment. **(D)** Western blot experiment demonstrates the efficacy of CREB antibody; the motifs of the top 4 CREB-binding genomic loci. **(E)** Percentage of CREB-binding genomic loci. **(F)** Treatment with CREB inhibitor for xenograft testicular models of DLBCL.

CREB is another vital regulator of tumor B cells, and we performed the experiment involving E2F to assess the activity of CREB. We added KG-501, a CREB inhibitor, to human B-lymphoma cells *in vitro* and found that the proliferation of cells was inhibited significantly and appeared to undergo apoptosis on day 3. The result of the CCK8 assay also showed that the cell proliferation capacity of the inhibitor group decreased on day 1 (Fig. 5A). In the CREB inhibitor group, cell morphology gradually began to change from day 1 to day 4. The cells were identified as apoptotic cells via annexin-PI/PE staining and flow cytometry on day 3. We found that 67.2% of cells in the Q3 quadrant could be identified as early apoptotic cells (Fig. 5B). In addition, the protein levels of cleaved caspase 3 were significantly higher than those of the control group, which confirms the above results (Fig. 5C). Chromatin immunoprecipitation sequencing was performed to identify the enriched loci of CREB (Fig. 5D). We found that CREB binding locations mainly occurred in the promoter (32.8%), intron (33.5%), and intergenic (26.46%) regions (Fig. 5D). Four CREB binding motifs (FAR1, SREBF1, SRSF2, and ZNF460) were found to be closely involved in human B-lymphoma cell proliferation (Fig. 5E). In addition, the CREB inhibitor exhibited specific functions in the primary testicular human B-lymphoma cell tumor model of server-combined-immune-deficiency mice. The results indicated that the testes in the “Tumor + CREB Inhibitor” group had smaller tumors compared with those in the “Tumor + DMSO” group but were slightly larger than those in the wild-type group. This suggests a potential reduction in tumor size attributable to the CREB inhibitor treatment, with sizes approximately ranging from 1 to 2 cm (Fig. 5F).

### Cell–cell interactions in normal testicular, paracancerous, and cancerous tissues

**Figure 6:**
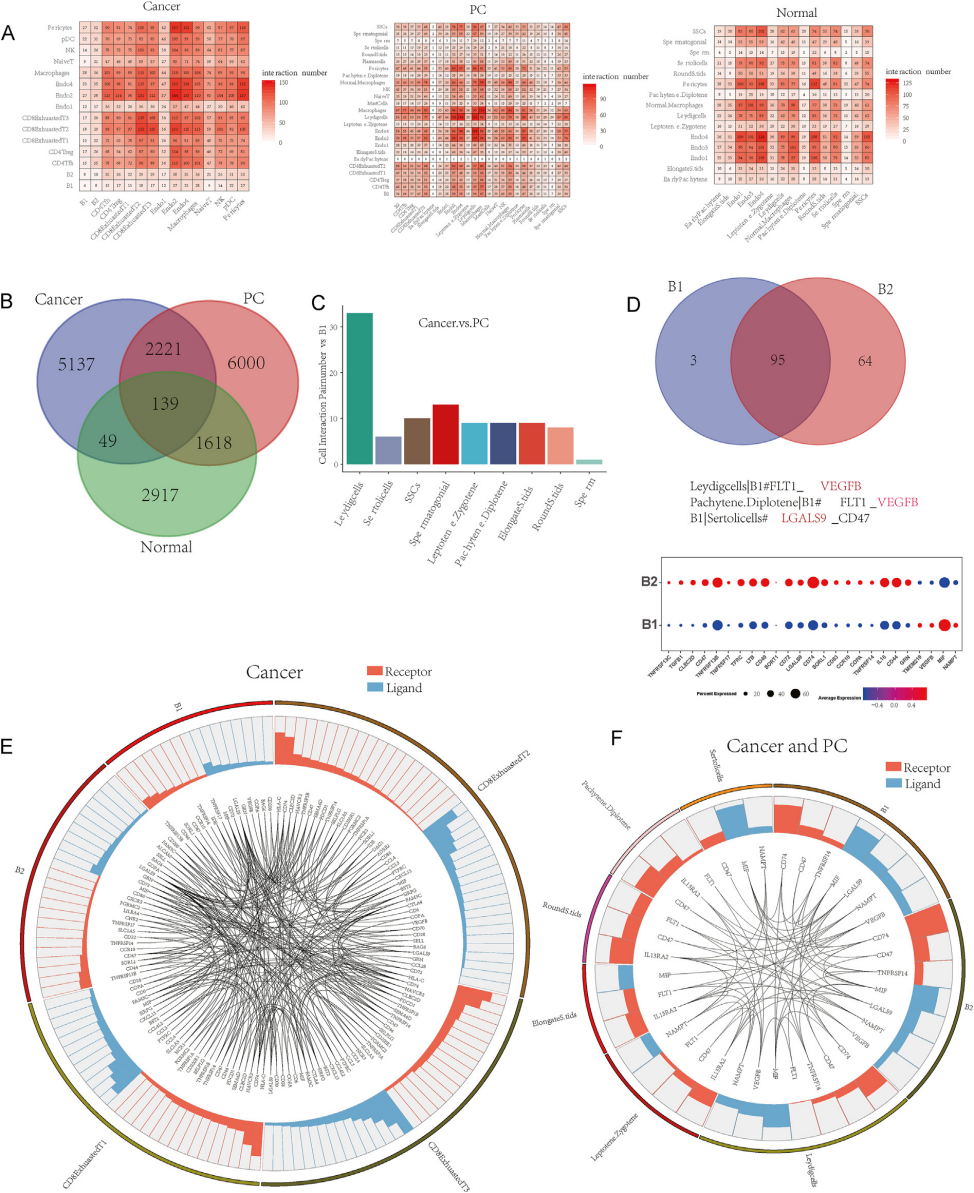
Cell–cell interactions in cancerous, paracancerous, and normal tissues. **(A)** Cell–cell interaction pairs in cancerous, paracancerous, and healthy tissues. **(B)** The Venn diagrams showing the number of genes expressed in cancerous, paracancerous, and normal tissues, and the genes expressed identically in different tissues. **(C)** Cell–cell interaction pairs of B1 and germ cells. **(D)** The Venn diagrams showing the number of cell–cell interactions gene pairs for tumor B cells and normal B cells with germ cells, respectively **(E)** Cell–cell interaction receptors and ligands between B1, B2, and CD8Tex1/2/3 cells in cancerous tissues. **(F)** Cell–cell interaction receptors and ligands between B1, B2, and germ cells.

Cross-talk between cancer cells and proximal immune cells ultimately leads to an environment that promotes tumor growth and metastasis.^46^ To understand the nature of these cell dialogs, we analyzed cell–cell interactions further. There were many intercellular interactions between CD8Tex1/2/3 and endothelial cells 1/2/4 in cancer tissues (Fig. 6A). The image in the middle of Figure 6A showed that cross-talk occurred between immune and somatic cells and the germ cells, which may induce a failure of spermatogenesis. In healthy tissues, endothelial cells frequently interact with germ cells, such as spermatogonial stem cells, and the pachytene and diplotene. Venn diagrams showed the number of gene interaction pairs in the three tissues (Fig. 6B). Paracancerous tissues exhibited the most gene interaction pairs, and those in cancer tissues were higher than in the healthy testis. Next, we analyzed the interactions of B1 cells with germ and somatic cells. We found that B1 cells mainly affected the Leydig and spermatogonia cells. Testosterone was almost entirely produced by Leydig cells, which played an essential role in spermatogenesis (Fig. 6C).^47^ B1 cells partially lost the ability to regulate with germ cells, but VEGFB, which was expressed at high levels, interacted with the Leydig cells, pachytene, and diplotene; this may affect the process of spermatogenesis (Fig. 6D). The relationship between B and exhausted CD8^+^ T cells was largely unknown. Here, we performed a preliminary analysis of the interactions between B1/2 and CD8Tex1/2/3 cells by assessing the receptors and ligands of these cells (Fig. 6E). The results showed that some B1 factors were strongly associated with CD8Tex1/2/3 cells. Recently, Giovannone et al reported that LGALS9 was autologously produced by naïve B cells and inhibited B cell activation via the suppression of calcium signaling.^48^ However, the relationship between B cells and T cells was largely unknown. Figure 6E showed that high levels of crosstalk occurred between normal B or malignant B cells and exhausted T cells. Finally, we analyzed the strong association between the receptors and ligands of cancerous and paracancerous tissues (Fig. 6F). These factors may affect the process of spermatogenesis in paracancerous tissues. These results demonstrated the cell–cell interactions in cancer and paracancerous tissues and malignant factors secreted by B cells that affected spermatogenesis and probably facilitated T-cell exhaustion.

### Spatial transcriptomics of PT-DLBCL tissues

**Figure 7:**
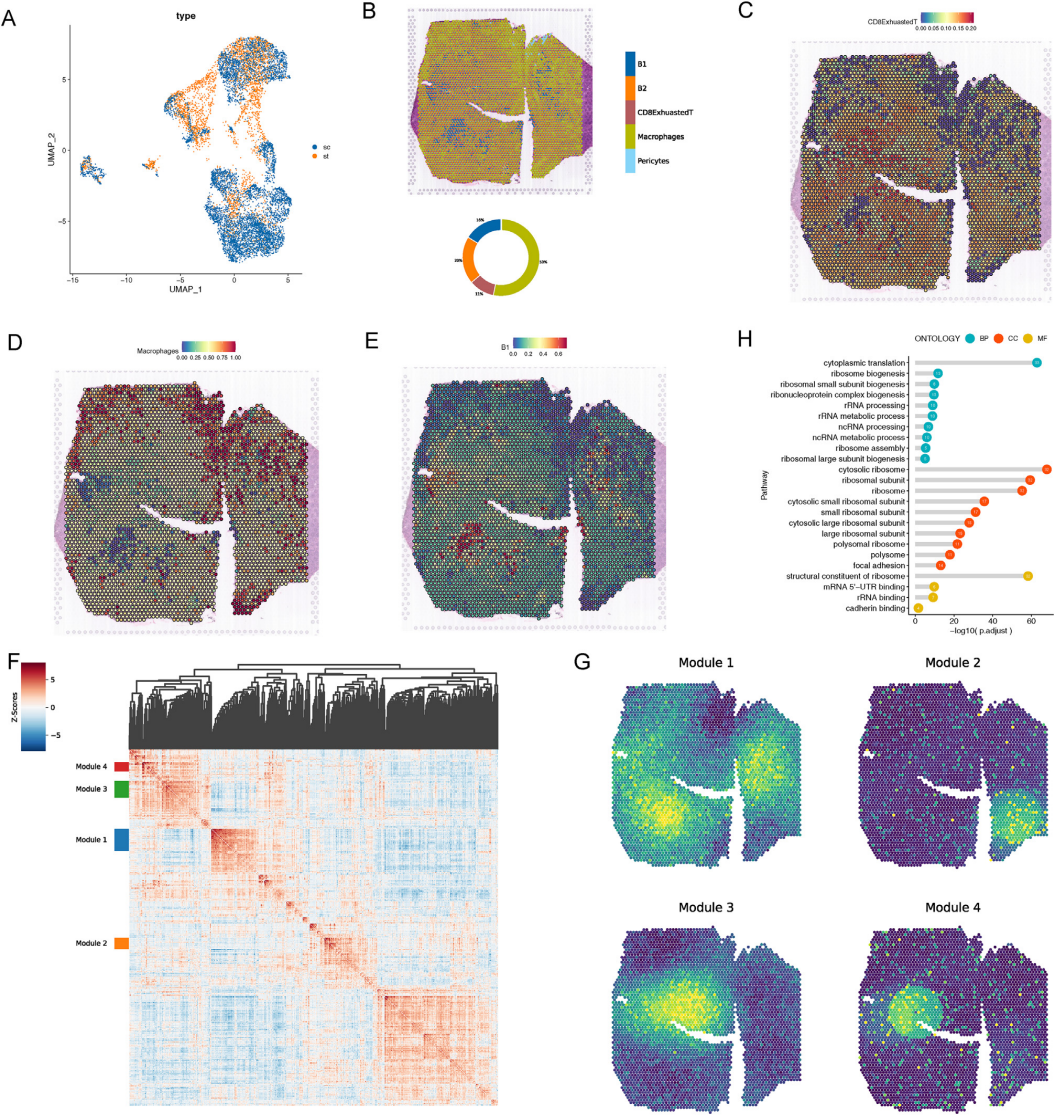
Spatial transcriptomics of PT-DLBCL tissue. **(A)** Co-embedding of spatial transcriptomic and single-cell RNA-sequencing datasets. **(B)** The spatial scatter pie plot representing the proportions of the cells from the reference atlas within capture locations in the carcinoma tissue. **(C**–**E)** Predicted proportion within each capture location for the cell types. **(F)** Genes with significant spatial autocorrelation are grouped into 4 gene modules based on pairwise spatial correlations. **(G)** Spatial gene modules are visualized with their summary by module scores. **(H)** GO analysis of Module 1 gene sets.

To generate spatial transcriptomic maps of PT-DLBCL sections, we mounted cryosections of the unfixed PT-DLBCL tissues onto spatially barcoded spatial transcriptomic microarray slides (see *Methods*). First, we conducted a deconvolution analysis of control spots to map out the cellular composition. The accompanying pie chart breaks down the cell type proportions, showing that exhausted CD8 T cells account for 11%, macrophages for 53%, B1 cells for 16%, and B2 cells for 20% (Fig. 7B). The result showed exhausted CD8 T cells densely populating most of the tissue section, suggesting a widespread immune response throughout the tumor environment (Fig. 7C). Macrophages predominantly cluster at the periphery of the tumor tissue. This distribution pattern suggests a potential role in the tumor's interaction with surrounding tissues and may indicate a tumor-associated macrophage response (Fig. 7D). B1 tumor cells are primarily clustered in the center of the tissue, indicating a focal point of tumor activity with a less dispersed distribution. However, exhausted CD8 T cells are spatially distributed around tumor B cells and this distribution pattern may be responsible for CD8 T exhaustion (Fig. 7E). Figure 7F presented a heatmap generated from the gene set analysis conducted with hotspot, which has identified four distinct gene modules within the dataset. Each module, marked by a unique color on the sidebar (Module 1 in blue, Module 2 in orange, Module 3 in green, and Module 4 in red), signifies a group of genes that are co-expressed and potentially co-regulated. Subsequently, these four modules were delineated within the tissue section, highlighting the distinct gene expression patterns characteristic of each module (Fig. 7G). Module 1 gene set shows a high expression pattern that corresponds closely with the B1 cell type spots. We performed an enrichment analysis of Module 1 genes, excluding ribosomal protein expression genes. The result, as shown in Figure 7H, indicates significant enrichment in pathways related to cytoplasmic translation, ribosome biogenesis, and various subunit processes. These findings highlight the biological processes and molecular functions that are predominantly active in Module 1, shedding light on the functional aspects of the B1 cell type spots that exhibit high expression of this gene module.

**Figure 8:**
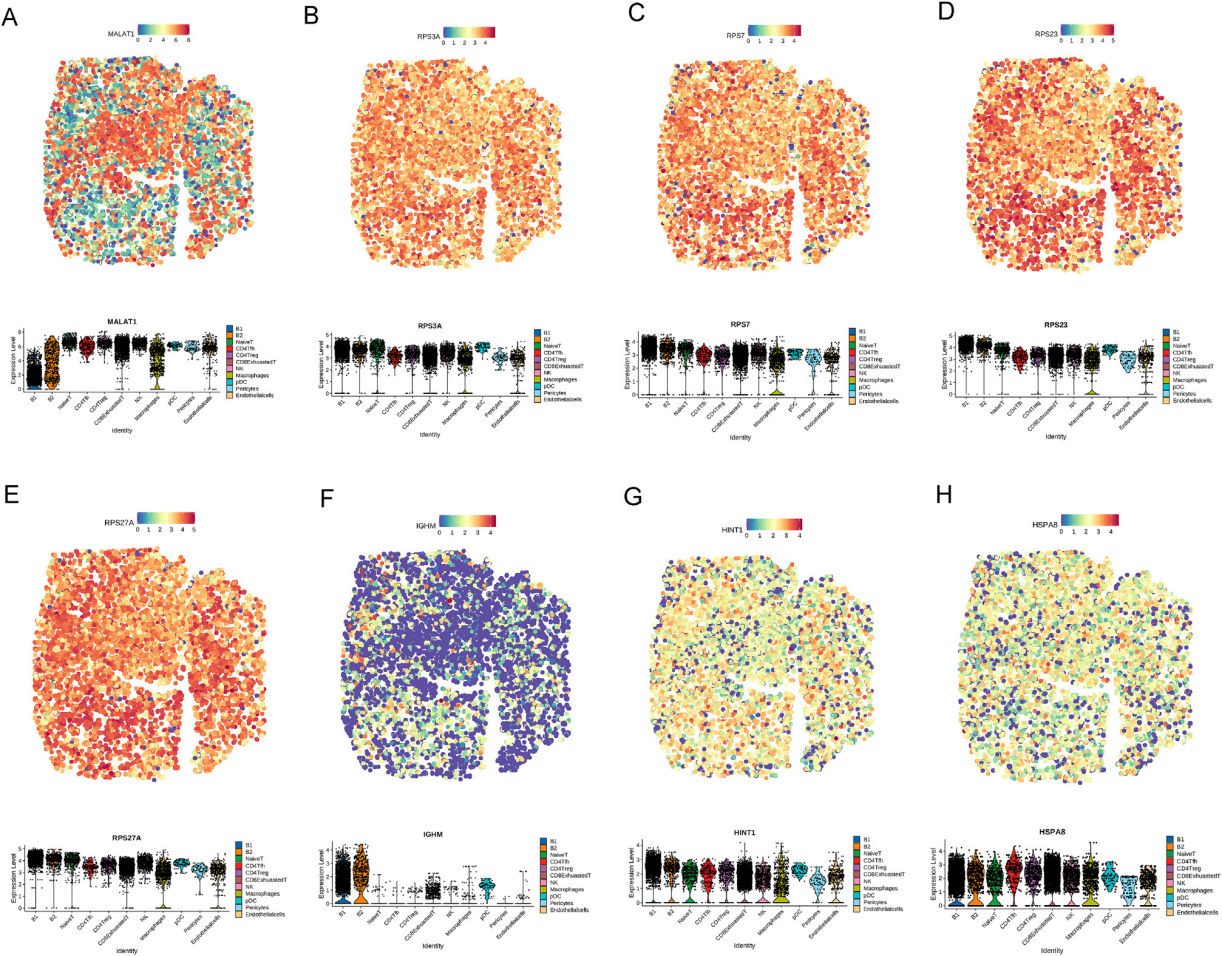
The spatial and single-cell RNA-sequencing expression patterns of key genes. **(A**–**H)** Representatives of differentially expressed genes in PT-DLBCL on spatial positioning and cell types. Above: each dot represents a spatially resolved data point, with the color intensity corresponding to the expression levels, as indicated by the color bar at the top of the figure. The expression levels range from low expression to high expression. Higher expression levels are shown in red, while lower expression levels are shown in blue. This spatial distribution highlights the heterogeneity of gene expression across the tissue sample, with distinct regions of high and low expression. Below: single-cell transcriptomics reveals gene expression across different cell types.

Next, we applied CellTrek to combine the scRNA-seq and spatial transcriptomic datasets to achieve single-cell spatial mapping (Fig. 7A) and identified the spatial location of key genes, including *MALAT1*, *RPS3A*, *RPS7*, *RPS23*, *RPS27A*, *IGHM*, *HINT1*, and *HSPA8*, which were expressed at high levels in different cell types. *MALAT1* was highly expressed in exhausted CD8^+^ T cells (Fig. 8A) and was reported to be linked to breast cancer, pancreatic cancer, prostate cancer, glioma, and leukemia.^49^ Notably, *RPS3A*, *RPS7*, *RPS23*, and *RPS27A* (Fig. 8B–E) were expressed at high levels in B1 cells than in other cell types. These four genes belong to the family of ribosomal proteins and are located in the cytoplasm. Wu et al reported that SMYD2 played a role in regulating RPS7, which promoted lung adenocarcinoma tumorigenesis and metastasis.^50^ High RPS3A expression levels were strongly associated with low tumor immune cell infiltration levels and an unfavorable prognosis in hepatocellular carcinoma patients.^51^ RPS27A was highly expressed in leukemia cells. It promoted proliferation, regulated cell cycle progression, and inhibited apoptosis.^52^ Our findings showed that HINT1 was highly expressed in B1 and exhausted CD8^+^ T cells (Fig. 8G). HINT1 encoded a protein that hydrolyzes purine nucleotide phosphoramidate substrates that play a role in tumor suppression. HSPA8, as a chaperone protein, facilitated the protein folding process and was expressed at high levels in acute myeloid leukemia patients (Fig. 8H).^53^ These results showed the spatial microenvironment of PT-DLBCL and the spatial distribution of key genes.

## Discussion

Primary testicular lymphoma is a rare and aggressive type of extranodal non-Hodgkin lymphoma, constituting less than 5% of all testicular cancers and representing 1%–2% of non-Hodgkin lymphoma occurrences. Approximately 80% of primary testicular lymphoma cases are diffuse large B-cell lymphomas. Population-based research has determined that the yearly incidence rate ranges from 0.09 to 0.26 cases per 100,000 individuals.^54^^,^^55^ Primary testicular lymphoma exhibits a pronounced extranodal tropism, with relapses often occurring in areas such as the central nervous system, skin, the contralateral testis, and pleura.^56^, ^57^, ^58^, ^59^, ^60^ However, the pathogenesis remains unclear. Here, we have combined scRNA-seq and spatial transcriptomics to identify distinct cell types, subpopulations, and cell states with normal, paracancerous, and cancer samples. We demonstrated that the microenvironment of PT-DLBCL was composed of T cells, B cells, macrophages, and endothelial cells. T cell exhaustion is a term that is widely used to describe the response of T cells to chronic viral infections, tumors, and chronic antigen stimulation. Hence, it is crucial to understand the features and pathways of T cell exhaustion in PT-DLBCL, as this would be helpful for the effective application of checkpoint blockades and adoptive T cell transfer therapies.^61^ Initially, exhausted CD8^+^ T cells were a uniform population but were recently reported to be composed of multiple interconnected subpopulations via a single-cell analysis.^62^ The results showed that three subpopulations of exhausted CD8^+^ T cells had different functions. Notably, CD8Tex2 cells failed to exhibit normal T cell functions. In addition, we identified some key genes and transcription factors that were strongly associated with the progression of exhausted CD8^+^ T cells and demonstrated that CD8 T exhaustion was a progressive process. Our study found a significant presence of CD8^+^ T cells in the TME and noted that the exhaustion of CD8^+^ T cells is a dynamic process. Future research focusing on the mechanisms of CD8^+^ T cell exhaustion could potentially enhance our understanding of the specific pathogenesis of PT-DLBCL and identify potential therapeutic strategies.

PT-DLBCL presents at an immune-privileged site of the testis with unique characteristics associated with NF-κB pathway signaling, tumor-infiltrating immune cells, and 9p24.1 aberrations, compared with other lymphoma entities.^14^ In humans, B cells develop from hematopoietic stem cells in the bone marrow and exhibit a highly rapid progression of the cell division process, including a series of somatic recombination and mutation events that enable the assembly of B cell antigen receptors. We analyzed the gene heterogeneity between malignant and normal B cells. B2 cells gradually differentiate into malignant B cells with changes in the gene expression pattern that may be strongly associated with this progression. Our results showed that malignant B cells account for ∼20% of the TME. In our results (Fig. 7B), B1 cells account for 16% and exhibit a clustered distribution. This may suggest that they accelerate tumor growth speed through a mechanism of distant metastasis.

The treatment of PT-DLBCL aimed to achieve complete remission and prevent a relapse of the contralateral testis and the central nervous system. However, because of the lack of randomized phase III trials, treatment recommendations mainly include surgical excision and chemotherapy.^63^ Recent studies reported that E2F or elevated E2F target expression was linked with poor prognosis in tumors such as liver and pancreatic cancers.^34^ Likely, E2F2 is essential for the maintenance of T lymphocyte quiescence. As a proto-oncogene, CREB has been reported to be involved in different tumor maintenance and progression. Thus, many CREB inhibitor compounds were developed and tested in different tumor cell lines over the past years.^14^ However, few studies reported the regulation relationship between E2F and CREB. Laresgoiti et al demonstrated that E2F2 and CREB cooperatively regulate the transcriptional activity of cell cycle genes. They found that activation of the transcription factor CREB DNA binding contributes to E2F2- mediated repression of Mcm5 and Chk1 promoters.^64^ Our results showed that E2F and CREB played an important role in the proliferation of malignant B cells. We also found that the down-regulation of E2F and CREB could inhibit human B-lymphoma cell proliferation and induce cell apoptosis. The E2F and CREB inhibitors could also prevent tumor progression in xenograft testicular models of DLBCL. We performed chromatin immunoprecipitation sequencing to clarify the regulation of E2F and CREB, and critical motifs were found. The down-regulation of these key critical motifs can aid in further understanding the roles and mechanisms of E2F and CREB in primary testicular lymphoma. This may assist in achieving the goal of inhibiting tumor growth by regulating E2F and CREB. In the future study, we will knock down or overexpress these genes in human B-lymphoma cells and further illustrate the regulation mechanism of E2F and CREB in PT-DLBCL.

The traditional treatment involved the surgical removal of the tumor side of the testes and irradiation of the contralateral testis.^65^ Patients fail to exhibit normal spermatogenesis and have substantial levels of psychological stress. Substantial progress has been achieved in primary testicular lymphoma treatment through the integration of radiotherapy, anthracycline-based chemotherapy, rituximab, and central nervous system-directed prophylaxis. While the regimen of R–CHOP every 21 days, complemented by intrathecal methotrexate and locoregional radiotherapy, is recognized as the global standard, there remains a gap in care for patients who do not respond to this protocol. In our study, we found that specific transcription factors, including VEGFB and LGALS9 that were secreted by malignant B cells affected germ cells in paracancerous tissues. Cancer development and progression occurred in tandem with alternations in the surrounding stroma. Cell–cell crosstalk through the secretion of various cytokines, chemokines, and other factors facilitated tumor growth and metastasis. The cells participating in crosstalk included macrophages, neutrophils, T cells, dendritic cells, innate lymphoid cells, B cells, myeloid-derived suppressor cells, and NK cells.^46^ We analyzed the cell–cell interactions between B2, malignant B1, and CD8Tex1/2/3 cells. VEGFB was probably a ligand of malignant B2 cells that targeted ADRB2 in CD8Tex2 cells, which in turn might have facilitated CD8 T exhaustion. In our study, scRNA-seq enables us to identify cell populations and TMEs in PT-DLBCL, but their spatial organization is yet to be characterized. We applied the 10× Visium spatial transcriptomics platform to identify the PT-DLBCL spatial transcriptome. Our findings demonstrated that identical types of cells show aggregated growth in the spatial transcriptome, but the expression patterns associated with the same gene were different. Components of key genes were highly expressed in B and exhausted CD8^+^ T cells.

Our study leverages spatial transcriptomics to showcase the spatial distribution of different cells within tumor tissues and the expression patterns of certain key genes. However, our research has still limitations. For instance, due to the rarity of this tumor tissue, we obtained only one sample. Current spatial transcriptomics technology does not achieve high resolution; thus, precise analysis of intercellular interactions is challenging. In terms of cellular mechanism research, we have only preliminarily demonstrated that inhibiting E2F and CREB has a suppressive effect on tumor cells, but the underlying mechanisms remain unclear and require further investigation. In addition, the lower modeling rate resulted in an uneven number of mice within the experimental groups, which may have affected the accuracy of the experiments to some extent. In subsequent research, we will increase the number of mice used for modeling to refine the study.

In conclusion, we combined scRNA-seq and spatial transcriptomics to reveal the TME and spatial organization of PT-DLBCL. Our findings showed that the TME was mainly composed of three subpopulations of exhausted CD8^+^ T cells and two subpopulations of B cells. Because of the limited number of patients, we could not systematically map the TME system, and we should include more samples in studies conducted in the future. We demonstrated that the down-regulation of E2F and CREB could inhibit human B-lymphoma cell proliferation *in vivo* and *in vitro* and elucidated the preliminary mechanisms of action using chromatin immunoprecipitation sequencing. Deepening our understanding of the pathophysiological mechanisms behind primary testicular lymphoma's distinct tropism offers the possibility of advancing treatments for this rare yet aggressive condition. Our study provides further insight into the tumorigenic progression mechanisms and potential novel therapeutic opportunities associated with PT-DLBCL.

## CRediT authorship contribution statement

**Xiaolong Wu:** Conceptualization, Data curation, Formal analysis, Writing – original draft, Writing – review & editing. **Jie Shi:** Formal analysis, Investigation, Methodology, Writing – original draft, Writing – review & editing. **Mujun Lu:** Data curation, Formal analysis, Investigation, Methodology. **Damin Yun:** Formal analysis, Investigation. **Sheng Gao:** Formal analysis, Investigation. **Longfei Hu:** Formal analysis, Software. **Fei Sun:** Conceptualization, Funding acquisition, Project administration, Resources, Supervision, Validation, Visualization, Writing – original draft, Writing – review & editing.

## Conflict of interests

The authors have no competing interests to declare.
