# Supplementary material for: The integration of single-cell RNA sequencing and spatial transcriptomics reveals the tumor microenvironment and spatial organization of testicular diffuse large B-cell lymphomas
Source: Genes Dis. 2024 Nov 30;12(4):101475. doi: 10.1016/j.gendis.2024.101475 (PMC12126957; doi:10.1016/j.gendis.2024.101475)
Supplement: Multimedia component 1 [file mmc1.docx]

**Supplemental Material and Methods**

**NaiveT cell identification and RNA Velocity trajectory analysis**

In order to distinguish between CD4 naiveT cells and CD8 naiveT cells, we defined naïve T cells that specifically express the CD4 gene as CD4naiveT cells, and those that specifically express the CD8A and CD8B genes as CD8naiveT cells, according to the expression of the CD4 and CD8 genes. We analysed CD4T cells and CD8T cells differentiation using the diffusion map embedding, separately. Diffusion map is a type of non-linear dimension reduction methods, and is recently applied to estimate the pseudo-time in single-cell transcriptomic data. In order to smoothly assess spliced and un-spliced mRNAs, we needed to convert the bam file to loom file by the function of run10x(function), provided by velocyto.py(function). Next, we merged multiple loom files by the function of loompy.combine (function) and then loaded the merged loom file into R software to combine analysis with the Seurat package. The result was projected to the Diffusion map plot for visualization consistency.

**Pseudotime and Clustering Analysis**

We explored the states and cell transitions of B cells in the testis by inferring the state trajectories using Monocle2. We used the ‘‘differentialGeneTest’’ function to derive DEGs from each cluster, and genes with a q-value < 1e-5 were used to order the cells during pseudotime analysis. After the cell trajectories were constructed, differentially expressed genes along the pseudotime were detected using the ‘‘differentialGeneTest’’ function. These genes were clustered using the “plot_pseudotime_heatmap”. Here, cells (in columns) were ordered by their pseudotime, and genes (in rows) were clustered via k-means clustering. Different kmean numbers were used to generate the optimal cluster number.

**Gene Regulon Analysis**

Regulon activity scores (RASs) of enriched transcription factors in each cell were derived using UMI matrix-based pySCENIC software. Command line implementation was achieved using pySCENIC software (1.10.0). The pyscenic grn command was used with the grnboos2 method, default options, and a fixed seed to derive co-expression modules between transcription factors and potential targets. The RAS matrices were then submitted to Seurat to investigate the regulatory networks underlying stromal cells. AUCell scores (regulon activities) of each cell were computed with the pycenic aucell command (default options). Notably, the number of target genes was highly variable from one regulon to another.

**Differential Gene Expression Analysis and Enrichment Analysis**

DEGs in a given cell type were compared to those in all other cell types using the FindAllMarkers function of the Seurat package (Wilcoxon rank sum test, p values adjusted for multiple tests using Bonferroni’s correction). All genes were probed for the computation of DEGs. If the genes were expressed in at least 10% of cells in either of the two compared populations, the difference in the expression on a natural log scale was at least 0.25. DEGs were filtered if |fold change| > 0.25 and FDR < 0.05. For differentially expressed genes in different cell types, genes with a p-value <0.05 and |logFC| > 0.25 were selected for enrichment analysis. The upregulated and downregulated genes were analyzed separately. GO and KEGG enrichment analyses were performed using ClusterProfiler (3.16.1).

**UCell Gene Set Scoring**

In order to demonstrate the degree of T-cell exhuasted, we have scored individual sub-populations of cells using functional gene sets. Gene set scoring was performed using the R package UCell(v1.1.0). UCell scores are based on the Mann-Whitney U statistic by ranking query genes’ in order of their expression levels in individual cells. Because UCell is a rank-based scoring method, it is suitable to be used in large datasets containing multiple samples and batches.

**Single Cell Copy Number Variations and Phylogenetic analysis**

To identify malignant cells with clonal large-scale chromosomal variations in copy number, we used the inferCNV R package to infer the genetic profiles of each cell, based on the average expression of large genes sets in each chromosomal region of the tumor genome, compared to that observed in healthy cells[31]. All B cells were categorized into the interrogation group, and T cells, NK cells, and plasma cells were sampled randomly as controls. Other parameters were set as default values. In order to export the linkage of copy number variation (CNV) events in different cell populations, we used the Minimal Event Distance Aneuploidy Lineage Tree (MEDALT) to infer the cell population lineage based on single cell copy number profiles. The genes that were significantly associated with lineage expansion were counted. We defined similar linkage associations as the amplification or deletion events associated with the same gene that occurred in different cells, and used a heatmap plot to show the CNV event association. Then, we further displayed gene expression levels in the genome region, gene amplification events in the red region, and gene deletion events in the blue region. We defined a threshold to indicate the degree of CNV variation, and showed that malignant cells exhibited gene expression levels that were higher than the threshold values.

**Cell-cell Interaction Analysis**

To analyze cell-cell interactions between different cell types, CellPhone DB was implemented to detect the interactions between cells based on normalized UMIs. The tool was run for 100 iterations and an expression threshold of 0.25 (limiting the analysis to genes expressed by 25% of cells). We focused on interactions between cancer cells and other cells for downstream analysis.

**Cell Culture and Treatment**

Human diffuse large B-cell lymphoma cell lines (DB) CRL-2289 were purchased from Procell Life (Wuhan, China). DB cells were cultured in RPMI-1640 medium (PM150110, Procell) containing 10% Fetal Bovine Serum (164210, Procell), 100 U/mL streptomycin, and 100 U/mL penicillin (PB180120, Procell) at 37 °C in a 5% humidified CO_2_ incubator. The CREB inhibitor (KG-501, Selleck) and E2F inhibitor (HLM006474, Selleck) were dissolved in dimethyl sulfoxide (D8371, Solarbio) and reposited at -80 °C (10 mM, stock solution). DB cells were cultured at a density of 3 × 10^5^ cells/well in 6-well plates. The cells were treated with 50 μM inhibitors at different times (0, 24, 48, 72, 96 h) and then collected for subsequent experiments.

**Cell Counting Kit-8 (CCK-8) Assay**

CCK-8 (A311, Vazyme) was used to detect cell viability. Cells transfected with inhibitors were resuspended in 1000 μL of medium and reseeded in 96-well plates in 100 μL of culture medium. Thereafter, the cells were incubated with 10 μL of CCK-8 reagent in each well and cultured for 2 h at 37°C. A microplate reader (BioTek, USA) was used to measure the optical density at 450 nm.

**Cell Apoptosis Assay**

The PE Annexin V Apoptosis Detection Kit I (559763, BD Bioscience) and Annexin V-FITC/PI Apoptosis Detection Kit (A211-01, Vazyme) were used for assessing apoptosis levels. Cells were cultured in 6-well plates and treated with KG-501 and HLM006474 (50 μM) for 72 h. After the cells were collected and washed thrice with pre-chilled PBS, they were stained using 5 μL Annexin V-PE and 7-AAD/PI per test for 15 min at room temperature in the dark. Ultimately, we used a flow cytometer (FACSCanto Ⅱ, BD) to analyze samples, and FlowJo was used for data analysis. Apoptotic cells were stained positively for PE Annexin V and stained negatively for 7-AAD.

**Western blotting**

Cells were harvested and washed thrice in PBS and lysed in a RIPA buffer (R0010, Solarbio) containing protease inhibitors to derive proteins. Twenty milligrams of total cell protein were loaded per sample, separated using 10% sodium dodecyl sulfate-polyacrylamide gel electrophoresis (SDS-PAGE), and transferred to a nitrocellulose membrane. The membrane was blocked with 5% non-fat milk in TBST (10 mM Tris, pH 7.5, 200 mM NaCl, and 0.2% Tween 20) for 1 h at room temperature. Then, membranes were incubated with primary antibodies at 4 °C overnight and washed thrice in TBST the following day. Subsequently, the membranes were incubated with horseradish peroxidase-conjugated anti-rabbit secondary antibodies (1:3000, ab216773, Abcam) for 2 h at room temperature. An Amersham Typhoon Fluorescent protein imaging system (GE Healthcare Life Sciences, Little Chalfont, UK) was used to visualize immunoreactive proteins using chemiluminescence.

**Real-Time** **Quantitative Polymerase-Chain-Reaction (RT-qPCR)**

Total RNA was extracted from samples using Trizol reagent (R1100, Solarbio) and purified using chloroform and ethanol. A cDNA kit (RR036A, TaKaRa) with reverse transcriptase was used to synthesize complementary DNA (cDNA) as the template for qPCR analysis according to the manufacturer’s protocol. The qPCR reaction mixture had 1.6 μL of a mixture of forward and reverse primers, 10 μL of TB Green mix (RR430A, TaKaRa), 2 μL of cDNA sample, and 6.4 μL of RNase-free water. Reactions were performed using a Light Cycler 96 Real-Time System (Roche, Switzerland) according to the manufacturer’s protocol. Each sample was run in triplicate wells. Thereafter, Ct values were obtained, and relative expression levels were determined using the 2-ΔΔCt method. β-actin expression levels were used for normalization. Statistical analyses were performed using GraphPad Prism (version 8, GraphPad Software Inc., San Diego, CA, USA). Statistical significance was measured based on whether *P < .05; **P < .01; or ***P < .001.

**10**× **Visium Spatial Transcriptomic Library Preparation**

The tumor sample was cut into 10 µm sections using Thermo Scientific CryoStar NX50 cryostat, and mounted on 10x Visium slides that were pre-cooled to −20 °C.We used the 10x Genomics Spatial RNAseq Visium platform for spatial transcriptomics experiments. A 10x Genomics Visium Gene Expression slide has 4 capture areas, each with an array of 5000 circular spots, containing printed DNA oligos for mRNA capture. The oligos on each spot have a PCR handle, unique spatial barcode, unique molecular identifier (UMI), and a poly-dT-VN tail for capturing the 3ˈ end of mRNA molecules. Each spot had a unique spatial barcode with diameter of 55 µm, and a center-to-center distance between spots of 110 µm. One 55 µm spot captured mRNA from 10 to 20 cells, depending on the cell size and packing density, which is variable across tissues. These sections were then fixed in pre-chilled methanol for 30 minutes, stained with H&E, and images were obtained. The 10x Genomics Cell Ranger software detects H&E-stained spots covered by tissues. The optimal permeabilization time for 10 µm thick tissue sections was found to be 12 min using the 10x Genomics Visium Tissue Optimization Kit. Spatially tagged cDNA libraries were built using the 10x Genomics Visium Spatial Gene Expression 3’ Library Construction V1 Kit. H&E-stained heart tissue sections were imaged using the Zeiss PALM MicroBeam laser capture microdissection system, and images were stitched and processed using Fiji ImageJ software. cDNA libraries were sequenced using Illumina NovaSeq output kits (Read 1 = 28, Read 2 = 120, Index 1 = 10, and Index 2 = 10). Fluidigm frames around the capture area on the Visium slide were aligned manually, and spots covering the tissues were selected using Loop Browser 4.0.0 software (10x Genomics). Sequencing data were then aligned to the human reference genome using the Space Ranger 1.0.0 pipeline to derive a feature spot-barcode expression matrix (10x Genomics).

**Chromatin Immunoprecipitation followed by sequencing analyses for transcription factors**

Chromatin Immunoprecipitation testing was performed using a SimpleChIP Enzymatic Chromatin IP Kit (9003, Cell Signaling Technology), and all experimental steps were carried out strictly following the manufacturer's instructions. Briefly, DB cells were crosslinked with formaldehyde and treated with ultrasound to cut the DNA into between 200 and 1000 bps segments. Equal cell lysates were precipitated overnight with antibodies at 4 ℃. The protein-DNA complex is reversed and purified to pure DNA the next day to prepare the ChIP-seq library. Subsequently, pair-end sequencing of the sample was performed on the Illumina platform (Illumina, USA). Library quality was assessed on the Agilent Bioanalyzer 2100 system. First, FASTP software is used to get clean data by removing reads containing adapters, reads containing ploy-N, and low-quality reads from raw data. The reference Genome and gene model annotation files are downloaded directly from the Genome website. After mapping reads to the reference genome, we used MACS2 peak call software to identify the background IP-enriched regions. Homer was used to detecting the de novo sequence motif and the matched known motifs. The genomic regions around the peaks were identified and annotated using ChIPseeker, then the functional enrichment results were identified by ontology (GO) enrichment analysis, and statistical enrichment of peak-related genes in the KEGG pathway was tested using KOBAS software.

**Software and algorithms used in this study**

| cellranger v3.0.2 | 10x Genomics | https://github.com/10XGenomics/cellranger |
| --- | --- | --- |
| Python version 3.7 | Python (2020) | https://www.python.org/downloads/release/python-370/ |
| R package v 3.16.1 | R Foundation for Statistical Computing (2017) | [https://www.R-project.org](https://www.r-project.org/) |
| Seurat (v3.1.2 R package) | Stuart et al., 2019 | https://github.com/satijalab/seurat |
| Harmony (v1.0, R package) | Korsunsky et al., 2019 | https://github.com/immunogenomics/harmony |
| ClusterProfiler (v3.16.1, R package) | Yu et al., 2012 | http://www.bioconductor.org/packages/release/bioc/html/clusterProfiler.html |
| CellPhoneDB(v2.1.0) | Efremova et al., 2020 | https://github.com/Teichlab/cellphonedb |
| UCell (v1.1.0, R package) | Andreatta et al., 2021 | https://github.com/carmonalab/UCell |
| Monocle2 (v 2.4.0 ) | Qiu et al., 2017 | https://cole-trapnell-lab.github.io/monocle-release/ |
| SynEcoSys | Yan Zhang et al.2023 | https://singleron.bio/products/synecosys/ |
| BayesSpace(v 1.2.0) | Zhao, E., Stone, M.R., Ren, X. et al. | https://github.com/edward130603/BayesSpace |
| Hotspot(v0.9.0) | DeTomaso D, Yosef N. | https://github.com/YosefLab/Hotspot |
| CellTrek(v 1.0.0) | Wei, R., He, S., Bai, S. et al. | https://github.com/navinlabcode/CellTrek |
| Scvelo(v 0.3.2) | Manno et al., 2018 | https://github.com/theislab/scvelo |
| pySCENIC(v 1.10.0) | Van de Sande B., Flerin C., et al. | https://github.com/aertslab/pySCENIC |
| inferCNV(v 0.1 ) | Anoop P. Patel, Itay Tirosh, et al. | https://github.com/broadinstitute/infercnv |
| MEDALT(v 1.0) | Wang, F., Wang, Q., Mohanty, V. et al. | https://github.com/KChen-lab/MEDALT |
| CellPhoneDB(v 2.0) | Efremova, M., Vento-Tormo, M., Teichmann, S.A. et al. | https://github.com/ventolab/CellphoneDB |

Results

The results showed that four endothelial cell subtypes (Endo1-4), indicating significant heterogeneity. In the tumor microenvironment, the endothelial cell subpopulations were primarily composed of Endo1 and Endo2 phenotypes. A significant prevalence of the Endo2 subtype was observed in pre-cancerous conditions, while this particular subtype was not detected within normal tissue samples (Fig.S1A-C). Subpopulations of immune cells were examined, with a particular focus on macrophages, plasmacytoid dendritic cells (pDCs), and mast cells. The umap plots depicted in Fig.S1D -E and illustrate the distribution of these immune cells across cancerous, pre-cancerous (PC), and normal tissues. Notably, a significant presence of pDCs was detected in the PC state, while pDCs were exclusively present in the cancerous, indicating their potential involvement in oncogenesis (Fig.S1D-F).

**Legends**

**Figure S1. scRNA-seq analysis of normal human testis, para cancerous, and cancer tissues obtained from a patient with testicular DLBCL.**

A) t-SNE plot showing the clustering of endothelial and cell subpopulations (Endo1, Endo2, Endo3, Endo4) within the sample.

B) t-SNE plot indicating the distribution of cell types in cancer tissue, with cancer cells, pre-cancerous cells, and normal cells annotated.

C) Proportion of endothelial cell subpopulations (Endo1, Endo2, Endo3, Endo4) in cancer, pre-cancerous, and normal tissue.

D) t-SNE plot displaying the clustering of immune cell types, including macrophages, plasmacytoid dendritic cells (pDC), and mast cells (Mast Cells).

E) t-SNE plot showing the overlay of cancer, pre-cancerous (PC), and normal cells with the immune cell distribution.

F) Bar graph depicting the proportion of immune cell types (macrophages, pDC, MastCells) in cancer, pre-cancerous, and normal tissue.

**Figure S2. CD4 T cells atlas of the testicular DLBCL microenvironment at single-cell resolution.**

A. The developmental trajectory of CD4 T cells is inferred by the diffusion map.

B. RNA velocity of each CD4 T cells subtype.

C. Heatmap illustrating the dynamics gene expression profile during CD8naiveT-to-CD8Tex differentiation.

D. Bubble plots depicting enriched biological processes (BP), cellular components (CC), and molecular functions (MF) for three distinct T cell subsets within PT-DLBCL patient tissue: CD4+ T follicular helper cells (CD4Tfh), CD4+ regulatory T cells (CD4Treg), and naive T cells (NaiveT).

**Figure S3. Wiggle plots analysis and qPCR for key genes**

(A) Bar graphs depicting the relative expression levels of SMAD3, MECOM, PAX8, and SFPQ in control and E2F inhibitor-treated samples.

(B) Wiggle plots of target genes in E2F.

(C) Bar graphs depicting the relative expression levels of FAR1, SREB1, SRSF2, and ZNF460 in control and E2F inhibitor-treated samples.

(D) Wiggle plots of target genes in CREB.
