# Supplementary figures and images for: The integration of single-cell RNA sequencing and spatial transcriptomics reveals the tumor microenvironment and spatial organization of testicular diffuse large B-cell lymphomas
Source: Genes Dis. 2024 Nov 30;12(4):101475. doi: 10.1016/j.gendis.2024.101475 (PMC12126957; doi:10.1016/j.gendis.2024.101475)

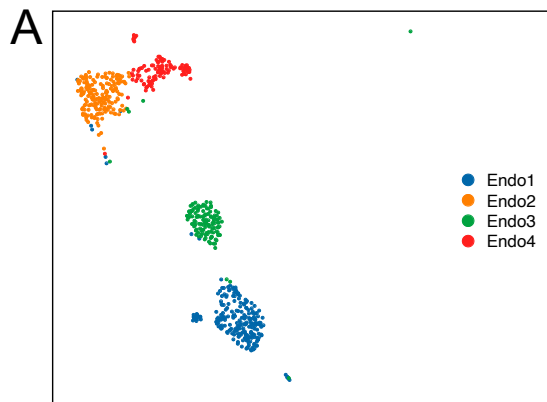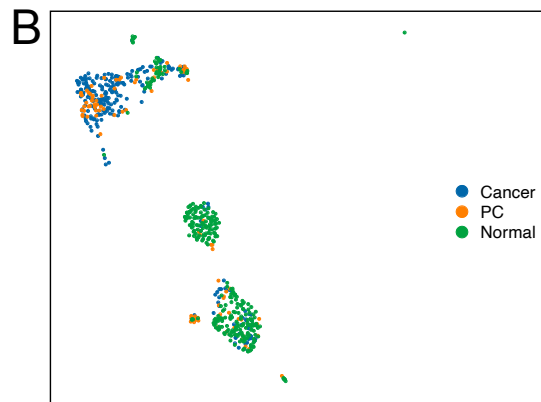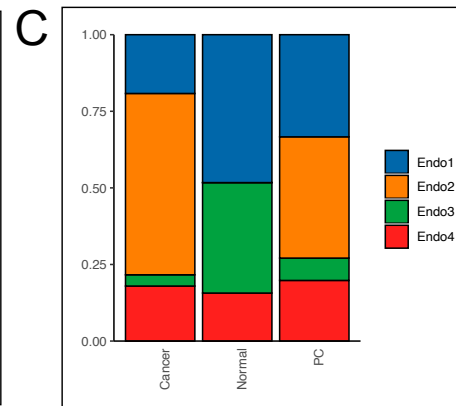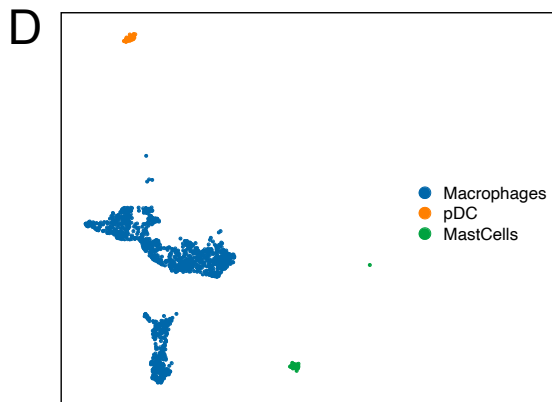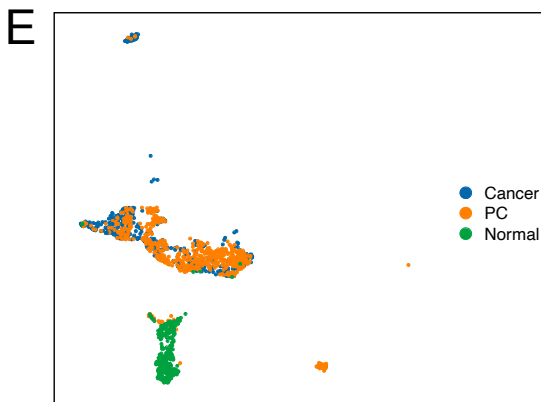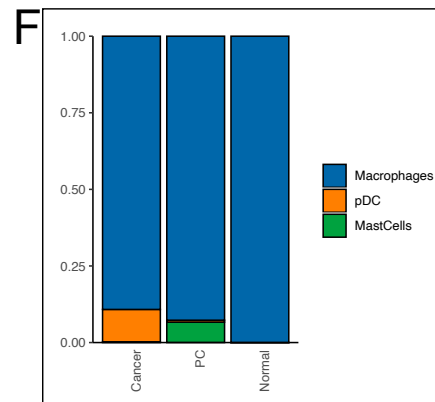

Supplement: Multimedia component 2 [file mmc2.pdf]

**A**

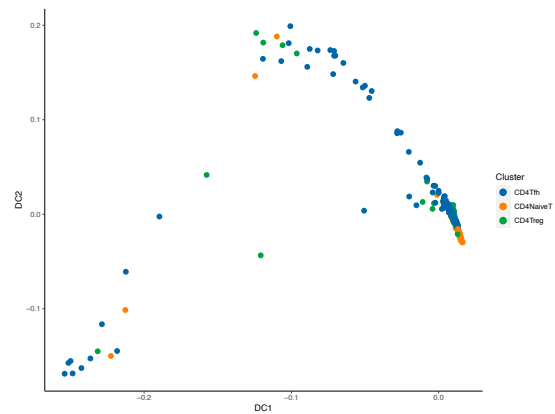

**B**

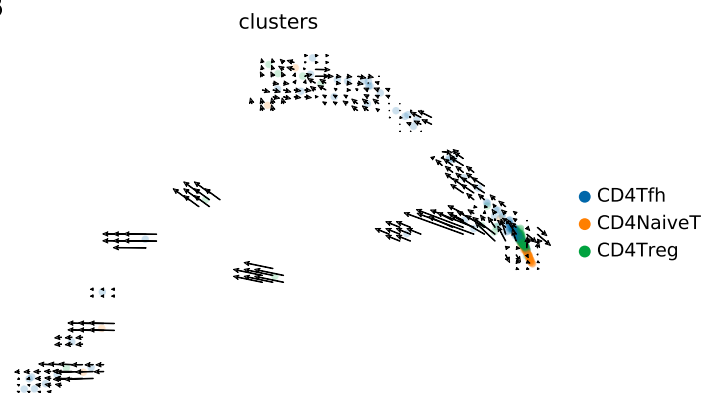

**C**

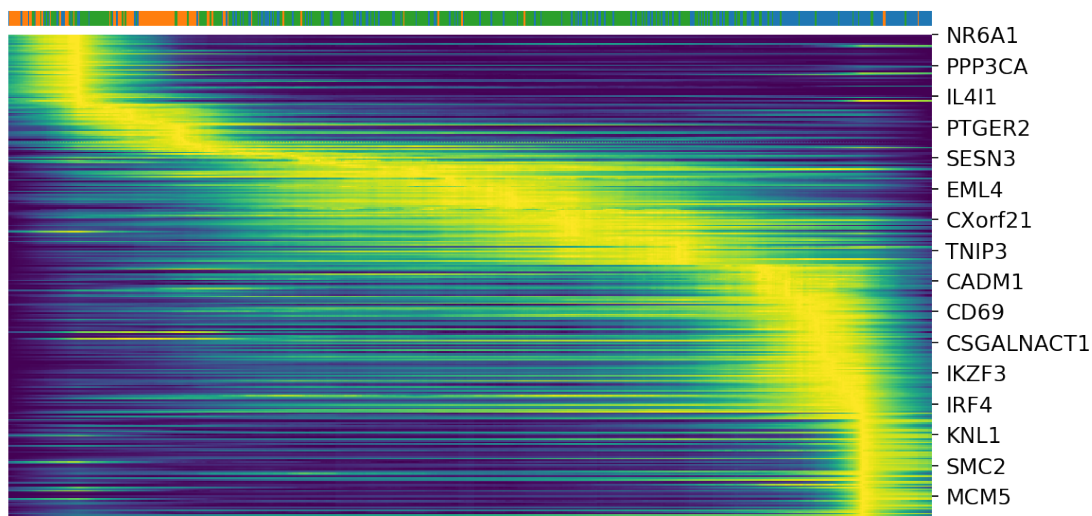

D

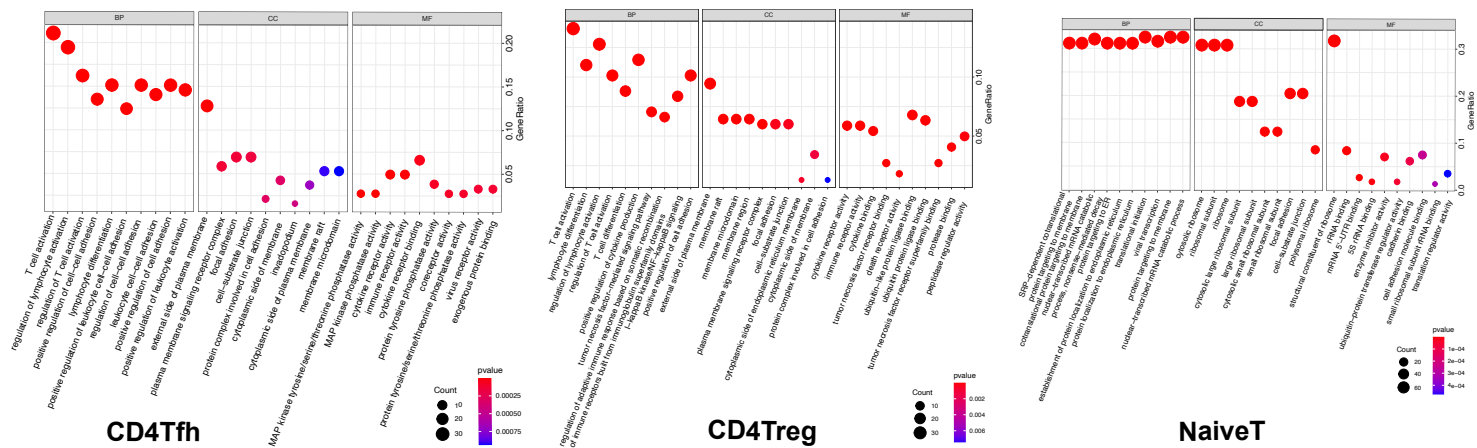

Supplement: Multimedia component 3 [file mmc3.pdf]

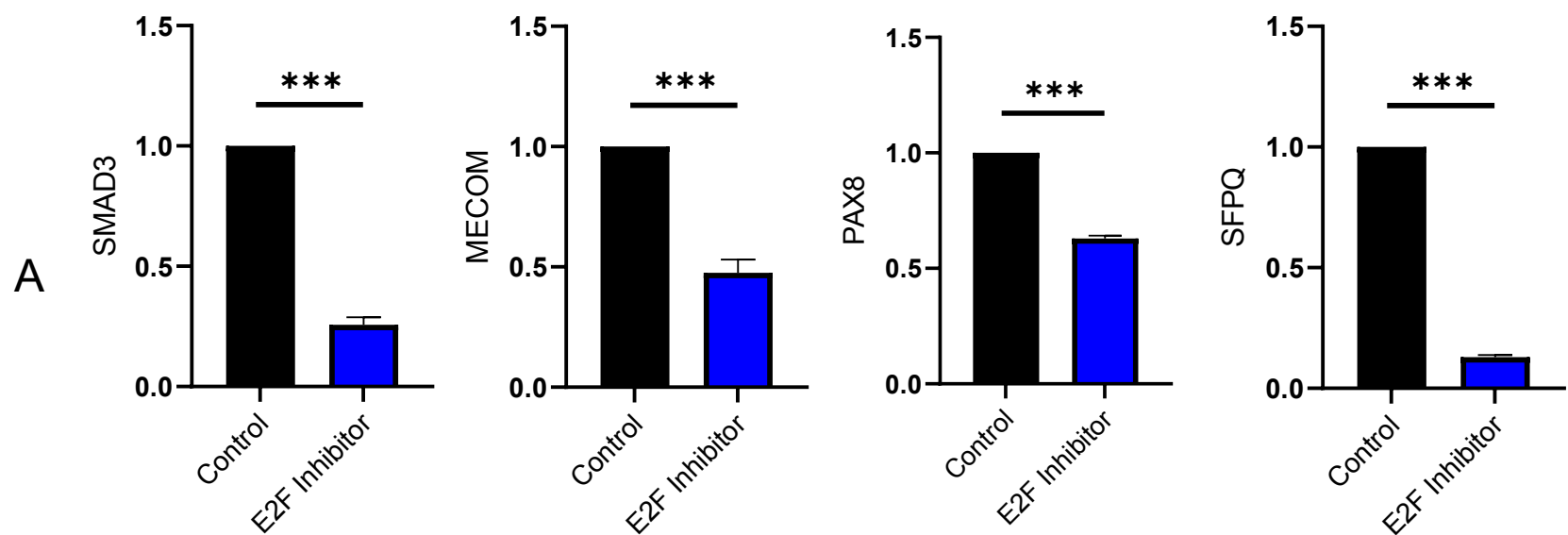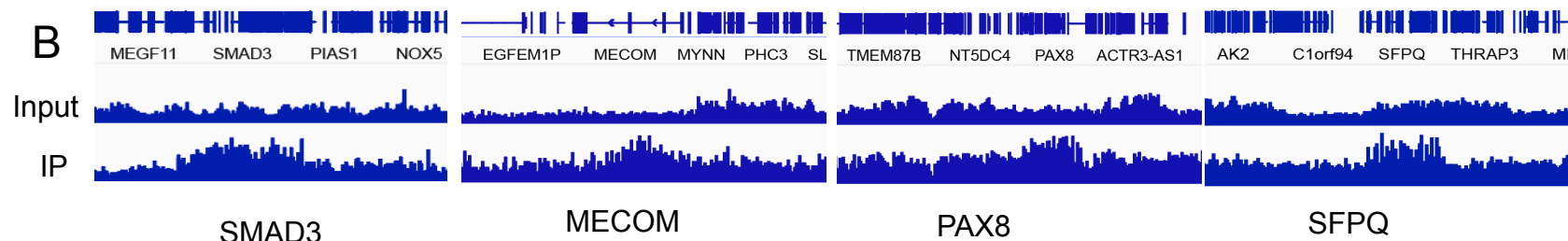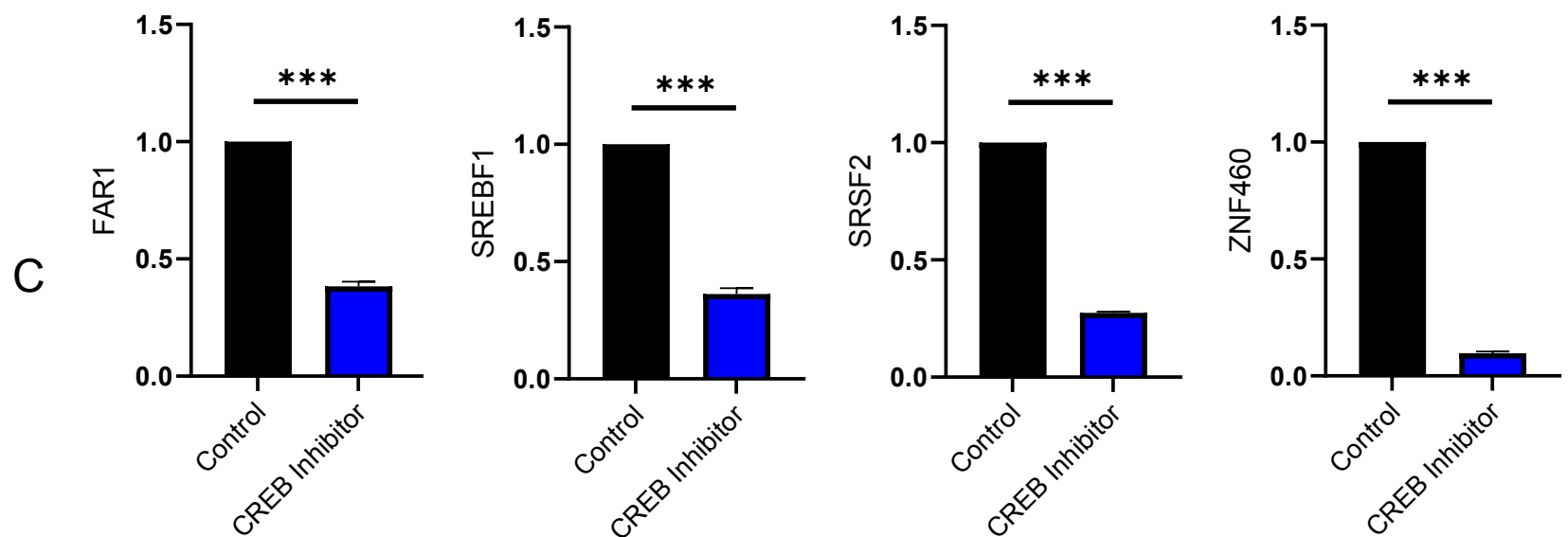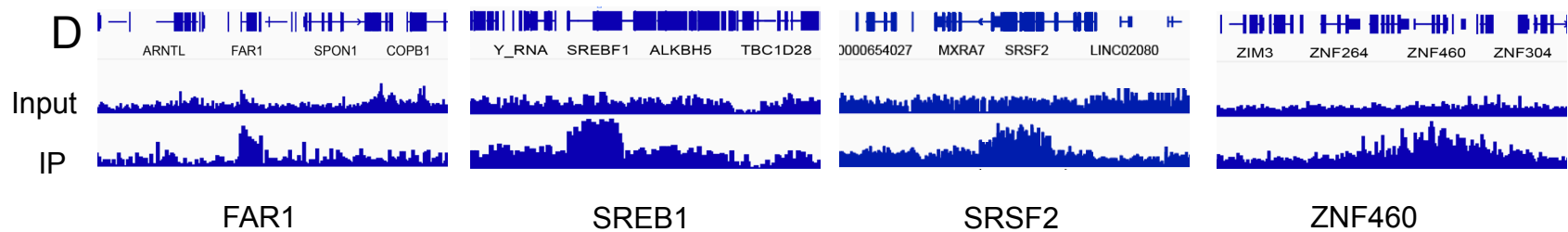

Supplement: Multimedia component 4 [file mmc4.pdf]
